# Foundation models and deep learning for cancer drug response prediction: a framework for data, metrics, and validation

**DOI:** 10.1093/bib/bbag225

**Published:** 2026-05-19

**Authors:** Katyna Sada Del Real, Vinay S Swamy, Josefina Arcagni, Eric Wang, Raul Rabadan, Angel Rubio

**Affiliations:** Departamento de Ingeniería Biomédica y Ciencias, TECNUN, Universidad de Navarra, Paseo de Manuel Lardizábal 13, 20018 San Sebastián, Gipuzkoa, Spain; Department of Biomedical Informatics, Columbia University, 622 West 168th Street, Washington Heights, New York, NY 10032, United States; Departamento de Ingeniería Biomédica y Ciencias, TECNUN, Universidad de Navarra, Paseo de Manuel Lardizábal 13, 20018 San Sebastián, Gipuzkoa, Spain; Google DeepMind, 1600 Amphitheatre Parkway, North Bayshore, Mountain View, CA 94043, United States; Google DeepMind, 1600 Amphitheatre Parkway, North Bayshore, Mountain View, CA 94043, United States; Departamento de Ingeniería Biomédica y Ciencias, TECNUN, Universidad de Navarra, Paseo de Manuel Lardizábal 13, 20018 San Sebastián, Gipuzkoa, Spain; Instituto de Ciencia de Datos e Inteligencia Artificial (DATAI), Universidad de Navarra, 31080 Pamplona, Spain

**Keywords:** cancer drug response, machine learning, metrics, framework

## Abstract

The emergence of large-scale omics data and foundational models has renewed efforts in the field of cancer drug response prediction (DRP). Despite recent progress, challenges such as limited tumor heterogeneity in standard cell lines and inconsistencies in experimental protocols across studies persist. However, these challenges also open significant opportunities for innovation. The complex nature of drug responses, influenced by variations in new patients and new drugs, presents a critical area for advancing validation approaches that traditional machine learning approaches often overlook. This review provides a comprehensive overview of the current state of DRP using advanced machine-learning models, discussing data sources, model designs, and evaluation methods. We introduce a unified framework for testing these models with a focus on clinically relevant metrics. By evaluating a range of foundational and deep-learning models within this framework, we identify performance gaps and propose concrete strategies to advance these computational models for reliable use in personalized cancer treatment, thereby unlocking their full clinical potential.

## Introduction

Cancer continues to account for millions of deaths annually, highlighting the urgent need for precision oncology—tailoring therapies to each patient’s tumor molecular profile to maximize efficacy and minimize toxicity. Central to this mission are machine-learning (ML) models that predict the likelihood that a specific drug will benefit an individual. Such *in silico* predictions guide oncologists in selecting effective treatments and help pharmaceutical researchers prioritize promising drug candidates, reducing costly late-stage trials. Currently, these drug response prediction (DRP) models are predominantly trained and validated using human cancer cell lines and animal models, with the goal of translating the most successful algorithms into clinical studies and ultimately routine patient care.

**Figure 1:**
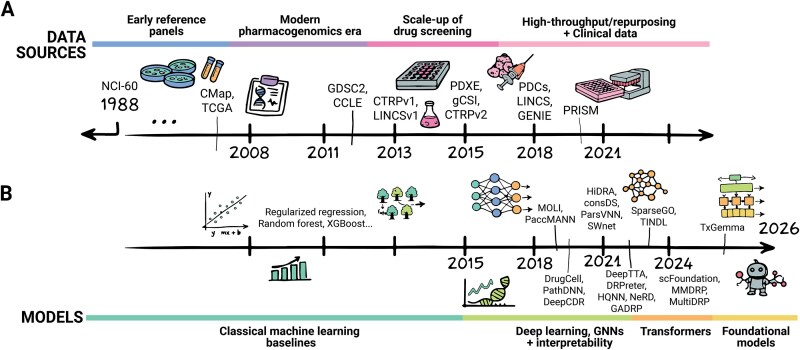
Data sources and modeling advances for cancer drug response prediction. (A) Timeline of key drug-response data sources, from early cell-line panels to large-scale pharmacogenomic screens and patient-derived models supporting precision oncology. (B) Timeline of modeling approaches for drug response prediction, showing the transition from classical machine learning to deep learning, graph-based methods, transformers, and emerging foundation models.

As shown in Fig. 1, the field has seen rapid advances in artificial intelligence (AI) and computational capabilities, enabling increasingly sophisticated DRP models. Deep learning has shown significant gains over traditional ML approaches [1]. Figure 1A traces the evolution of data resources from early reference panels (NCI-60 [2–5], 1988) through the emergence of large-scale pharmacogenomics databases (CCLE [6, 7], GDSC [8–10], LINCS [11]), patient-derived models (PDCs [12], GENIE [13]), and most recently high-throughput clinical datasets (PRISM) [14]. Figure 1B mirrors this progression algorithmically, from classical regularized regression and ensemble methods, through deep learning architectures (DeepCDR [15], MOLI [16], PaccMann [17]), and finally transformer-based foundation models (scFoundation [18], TxGemma [19]).

More recently, attention in the field has shifted toward foundation models (FMs)—large AI systems pre-trained on massive, diverse datasets—which can be adapted to multiple biomedical tasks without extensive task-specific data or retraining [20]. These models represent a paradigm shift, offering new approaches to data integration, generalization, and knowledge discovery, with success in target identification, gene–disease association, pharmacokinetics, miRNA–target interactions, CRISPR repair, and drug response prediction [19].

In this review, we examine the current landscape of ML for DRP, focusing on data sources and evaluation strategies. These models rely on diverse patient-derived characteristics, including multi-omics and lifestyle data [21]. Resources such as the Cancer Cell Line Encyclopedia (CCLE) and The Cancer Genome Atlas (TCGA) provide genomic information from sequencing technologies. Integrating heterogeneous omics datasets is challenging, requiring sophisticated models and a nuanced understanding of biological interactions [22]. Ensuring data quality, standardizing pipelines across laboratories, and reconciling differences between technologies such as RNA-seq and microarrays is crucial for reproducible and generalizable models, and addressing these issues creates opportunities for innovation in multi-omics integration, harmonization, and normalization.

Another major limitation is the scarcity and incompleteness of drug sensitivity data, especially when compared with abundant genomic datasets. This limitation, together with reliance on simplified models such as cancer cell lines, restricts the ability of current models to capture tumor heterogeneity and complexity [23, 24]. Patient-derived systems—including patient derived cell cultures (PDCs), patient derived organoids (PDOs), and patient derived xenografts (PDXs)—offer more physiologically relevant representations [12, 16, 25, 26]. Nevertheless, each system has constraints, such as absence of a human immune component, high costs, and ethical concerns. While subcutaneous PDXs are easier to monitor, orthotopic PDXs more accurately replicate tumor microenvironments [27].

Despite these challenges, opportunities for advancement are emerging. Data scarcity has driven innovation in computational approaches such as transfer learning, leveraging *in vitro* data to generalize to complex biological systems. Organ-on-a-chip [28] and microfluidic technologies [29, 30] offer promising *ex vivo* platforms, bridging simplified and *in vivo* models. Global initiatives to build shared repositories of patient-derived models and drug response data will be pivotal. Foundation models complement these efforts by pretraining on vast biological corpora, enabling knowledge transfer to DRP tasks and robust performance even with limited response data.

**Figure 2:**
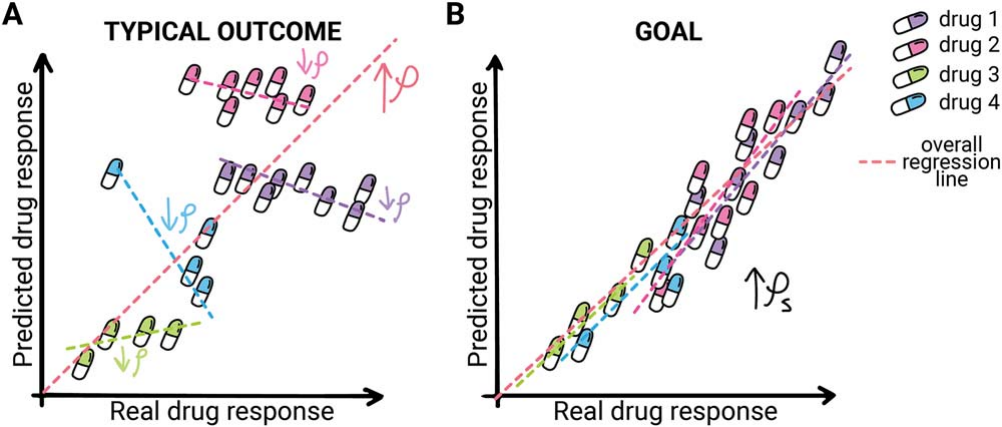
Scatter plots of a model trained on multiple drugs. This figure compares the performance of drug response prediction models across different scenarios. (A) Shows a model that has a strong overall correlation but performs poorly when measuring correlation for each drug. (B) Illustrates the desired outcome where the model’s predictions closely match actual drug responses.

A critical shortcoming in DRP model development lies in inadequate validation practices. Standard k-fold cross-validation, which randomly distributes all drugs and patients across training and testing sets, overestimates performance and does not reflect real-world conditions. Clinically meaningful validation paradigms—cell-blind and drug-blind—test generalization to unseen contexts. Additionally, reliance on overall correlation metrics can obscure poor performance for individual drugs. As shown in Fig. 2A, a model may appear accurate overall yet fail for specific drugs due to biases toward general properties, such as toxicity [31]. Incorporating per-drug and per-cell line metrics alongside overall scores better captures model strengths and weaknesses.

Interpretability is increasingly recognized as essential in clinical settings, where understanding predictions is key to adoption. Achieving interpretability remains challenging, particularly for deep learning models. In our previous work [32], we addressed this by developing an interpretable DRP model using visible neural networks (VNNs) [33], embedding domain knowledge directly into the architecture. While promising under rigorous cross-validation, recent findings reveal ongoing limitations across DRP models [16, 24, 31, 34], particularly due to the lack of standardized benchmarking frameworks [25].

To address these limitations, this review provides a comprehensive analysis of the field. We detail DRP model development, including data sources, cancer and drug representation strategies, model architectures, and evaluation methodologies. We also highlight challenges in comparing drug response metrics such as IC50 or AUDRC across datasets with differing protocols and concentration ranges.

We propose a standardized framework for DRP models emphasizing cross-validation techniques designed to simulate real-world scenarios. To support practical adoption, we introduce ai4clinic, a Python package offering tools for streamlined cross-validation and per-drug evaluation. Within this framework, we benchmark four representative architectures—SparseGO, DeepCDR, DeepCDR+scFoundation, and TxGemma—spanning classical deep learning, omics-specific foundation models, and general-purpose large language models. This comparison systematically evaluates how design choices impact predictive performance and clinical utility.

This manuscript has two complementary components. First, we provide a comprehensive review of the DRP landscape (data sources, drug representations, and model architectures sections), synthesizing current approaches and identifying critical gaps. Second, we present a focused benchmarking study (benchmarking section) that evaluates four representative architectures under clinically relevant validation scenarios using our proposed ai4clinic framework. This dual approach allows us to both contextualize the field and demonstrate practical implementation of standardized evaluation practices.

Unlike previous reviews that focus primarily on cataloguing model architectures or data repositories [23, 35], our work makes three distinct contributions. First, we provide systematic analysis of foundation models in DRP—an emerging paradigm that has not been comprehensively reviewed in this context. Second, we introduce a standardized evaluation framework (ai4clinic) with rigorous validation protocols that address critical limitations in current benchmarking practices, particularly the overuse of random splitting that inflates performance estimates. Third, we present focused benchmarking experiments comparing interpretable VNN models with recent foundation models across three clinically relevant data-splitting scenarios, illustrating the practical impact of validation strategy choices on model performance and generalizability.

## Data sources: the models, the drugs, and the experiments

Precision oncology employs various strategies to improve cancer treatment outcomes. Drug–target interactions determine how medications impact specific targets, while drug–drug similarity selects compounds based on structural similarities. These methods have enhanced our understanding and treatment of cancer [1]. However, this study will concentrate on the drug response prediction problem.

Panels of cell lines, such as those in CCLE, have been characterized across various omics levels to support this effort. These panels, along with drug response profiles from datasets like the Genomics of Drug Sensitivity in Cancer (GDSC), are commonly used as training data for many predictive models [31]. Other common drug response datasets include the NCI60 (National Cancer Institute’s 60 human tumor cell lines), CTRP (Cancer Therapeutics Response Portal), PRISM (Profiling Relative Inhibition Simultaneously in Mixtures), and gCSI (Genomic Cancer Sensitivity Index) [36]. Additionally, patient drug response data can be accessed through the TCGA portal and PDXs from the PDX Encyclopedia.

**Figure 3:**
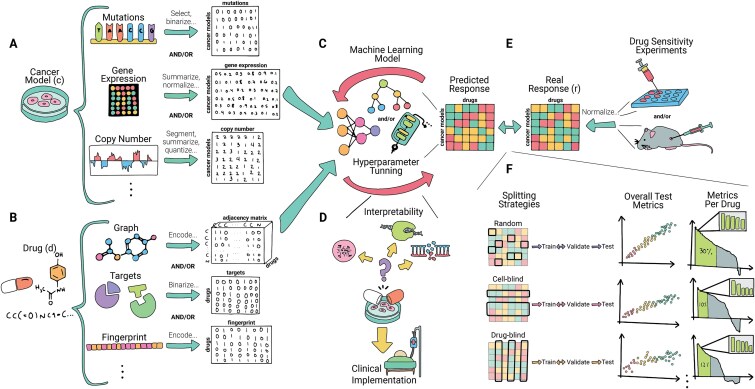
Overview of the drug response prediction framework. (A) The process begins with collecting clinical, genomic, and/or transcriptomic data from cell lines and/or patient-derived samples, along with their (E) drug sensitivity responses (real responses). (B) Drug representations, such as SMILES, molecular graphs, or fingerprints, are also identified. All omics, drug data, and responses are then normalized or encoded and integrated as features for a machine learning model. These datasets (C) train predictive models, which are (F) validated and tested by splitting the drug-cell pairs using different techniques: random, cell-blind (where the test set contains cell lines not in the training set), and drug-blind (where the test set contains drugs not in the training set), among others. Performance metrics are computed for all predictions and for individual drug predictions. (D) The models are designed to support clinical applications, emphasizing the importance of interpretability.

As illustrated in Fig. 3, DRP models normally require datasets with $N$ samples, structured as $S={\left\{c,d,r\right\}}_{i=1}^N$, where $c$ represents the cells or cancer models (e.g. cell line, PDC, PDO, or PDX), $d$ the drug, and $r$ the response indicating sensitivity to that drug, either continuous or binary [25]. Our objective is to establish a standardized framework that facilitates the effective development and comparison of models across datasets with this structure. This framework is applicable irrespective of the features used to represent the cells and drugs or the architecture of the ML model.

### Cancer models

For training DRP models, we require biological samples that represent cancer. These samples are primarily cancer cell lines, but can also include PDXs, PDCs, or organoids. As shown in Fig. 3, samples undergo thorough analysis and characterization to capture their molecular profiles. Several databases offer profiles of cells, with some concentrating specifically on cell lines, while others, such as CTRP and PRISM, only offer drug response data. Among these resources, CCLE stands out as a widely used database that has cataloged more than 1000 cell lines and even incorporates proteomic data. Table 1 provides an overview of these datasets, detailing key characteristics such as the number of profiled cells, those screened in drug sensitivity experiments, the types of omics data collected, and the technologies used for data acquisition.

**Table 1:** Overview of datasets used in drug response studies.

Dataset	Cells profiled	DNA mutation	mRNA expression	DNA copy number	DNA methylation	Drugs	CellS screened	Drug responses	Viability assay	Citation
NCI60^a^	60	Agilent WHG Illumina WXS	Affymetrix HG-U95, HG-133, HG-U133 Plus 2.0, Exon 1.0 ST array	Agilent 44K, NimbleGen 385K, Affymetrix 500K, Illumina Human1Mv1_C	Illumina Human Methylation450	> 100 000	60	≈ 4 000 000	Sulforhodamine B	[2–5]
CCLE	≈1000	Illumina Targeted sequencing, WGS, WXS	Affymetrix HG-U133 PLUS 2.0, Illumina RNAseq	Affymetrix SNP 6.0	Bisulfite sequencing (RRBS-seq)	24	479	≈ 11 000	CellTiterGlo	[6, 7]
GDSC I	≈1000	Illumina WXS	Affymetrix HG-U219	Affymetrix SNP 6.0	Illumina Human Methylation 450	403	970	333 292	Resazurin or Syto60	[8, 9]
GDSC II	≈1000	Illumina WXS	Illumina RNAseq	Affymetrix SNP 6.0	–	297	969	243 466	CellTiterGlo	[9, 10]
CTRP I	–	No	No	No	No	185	242	≈ 40 000	CellTiterGlo	[65]
CTRP II	–	No	No	No	No	481	860	≈ 300 000	CellTiterGlo	[66]
gCSI	675	Illumina WXS	Illumina RNAseq	Illumina Human Omni arrays	No	16	410	≈ 50 000	CellTiterGlo	[67, 68]
PRISM	–	No	No	No	No	>8000	>900	≈ 700 000	PRISM Repurposing Assay	[14]
PDCs (EGAD00001003441)	462	Illumina WXS	Illumina RNAseq	Illumina WXS	No	60	≈ 90	≈ 5000	ATPlite 1step;	[12]
LINCS	248	No	L1000 (978 landmark)	No	No	33 609	248	>1 300 000	Luminex (PRISM), Live-cell imaging (GR metric), ATPlite	[11]
CMap	9 (Core) 98 (Total)	No	Affymetrix HG-U133A	No	No	1309	5	7000	None	[39]

This table summarizes their key characteristics, including the number of cells profiled, types of omics data collected (DNA mutation, mRNA expression, DNA copy number, and DNA methylation), the number of drugs and cells screened in drug sensitivity experiments, and the viability assays employed.

^a^In the case of NCI-60 it is possible to get sequencing information from other sources.

Genomic data plays an important role in understanding the mechanisms of drug responses in cancer. This includes data on gene mutations, gene expression profiles, copy number variations (CNVs), and DNA methylation patterns. Each of these data types provides a unique perspective on the genetic factors that influence how cancer cells respond to different drugs. For instance, mutations in specific genes can activate or inactivate signaling pathways, affecting drug sensitivity. CNVs can lead to an overexpression or underexpression of certain proteins, which may impact drug efficacy. Gene expression profiles reveal the activity levels of genes under various conditions, and methylation patterns can indicate which genes are turned on or off. Different studies have shown [23, 36–38] that when integrating these genomic data, DRP models can gain a more comprehensive understanding of the genetic landscape of cancer and predict drug responses with greater accuracy. Traditionally, data extracted from DNA—such as mutations, CNVs, and translocations—are more straightforward to implement in clinical practice compared to gene expression or proteomics data.

Further details on data preprocessing and integration procedures, including normalization, feature selection, and batch correction strategies applied across datasets (e.g. CCLE, GDSC, and TCGA), are provided in the Supplementary Methods. In addition to cell line panels, large-scale transcriptional signature databases provide complementary resources. The Library of Integrated Network-based Cellular Signatures (LINCS) [11] and Connectivity Map (CMap) [39] offer extensive transcriptional response profiles following drug perturbations, enabling signature-based drug response prediction and repurposing studies. These resources capture dynamic cellular responses and have proven valuable for identifying drug mechanisms of action.

### Drugs parametrization

General-DRP models incorporate both cell line features and drug features, allowing them to predict responses for any drug using the same model. For them, accurate drug representation is essential for capturing molecular structure and properties in a machine-learning–friendly format [26]. Common approaches include linear notations (e.g. SMILES, InChI, and SELFIES), molecular fingerprints (e.g. MACCS, Morgan, and ESPF), graph-based structures, deep learning embeddings, and drug-target features. Each method offers different trade-offs between interpretability, structural resolution, and computational efficiency [40].

Table 2 summarizes representative DRP models and their corresponding drug representations. Detailed descriptions of encoding strategies, feature extraction methods, and specific examples of DRP models employing each type of representation are provided in the Supplementary Methods.

**Table 2:** Overview of DRP models.

Reference	Architecture	Omics	Omics source	Drug	Response	Split types	Overall metrics	Metrics per drug
TxGemma [19]	LLM	Cell line name and description	GDSC1 and GDSC2	SMILES	ln(IC50) GDSC1 and GDSC2	R	PCC	None
scFoundation [18]	Transformer	GE	CCLE	Molecular graph	ln(IC50) GDSC1	R, leave-one-drug-out, leave-one-cell-line-out	PCC	PCC
XGDP [69]	Graph CNN	GE	CCLE	GNN	IC50 GDSC	R, DB	RMSE, PCC, R^2^	None
MMDRP [38]	GNN	Multi (8 types, e.g. mRNA, CNV, RPPA)	CCLE	Molecular graph	AADRC CTRPv2	CB, DB, CTB, B	MAE, RMSE	None
nest [47]	VNN	Mut, CN	CCLE	None	AUDRC + Binarized AUDRC CTRP and GDSC	DB not applicable	-	PCC, diagnostic odds ratio
DeePAEG [56]	Graph CNN	Multi (GE, Mut, Meth, CN)	CCLE	ESPF of SMILES	ln(IC50) GDSC	R	PCC, SCC, RMSE (and per cancer type)	PCC, SCC, RMSE
Glioma DNN [45]	DNN	Multi (GE, Mut)	GDSC	None	IC50 + Binarized IC50 GDSC	R, DB not applicable	-	ACC, SEN, SPC, PRE, F1, FPR, GM, MCC
HQNN [70]	Quantum NN	Mut	GDSC	Molecular graph	IC50 GDSC	R	RMSE	None
MTIGCN [46]	Graph CNN	GE	GDSC and CCLE	DL embedding of PubChem FP	IC50 + Binarized IC50 GDSC and CCLE	R, CB, DB, M	PCC, SCC, RMSE; AUPRC (for binary task)	None
TINDL [24]	DNN	GE	GDSC	None	ln(IC50) GDSC	R, DB not applicable	-	PCC, *P*-value (for TCGA)
SparseGO [32]	VNN	Mut or GE	GDSC1, GDSC2, CTRPv2 and CCLE	Morgan (2048, r = 2)	AUDRC GDSC1, GDSC2 and CTRPv2	R, CB, DB	PCC, SCC, RMSE	PCC, SCC
DeepTTA [37]	Transformer + NNs	GE	GDSC2	ESPF of SMILES	ln(IC50) GDSC2	R, CB, DB, CTB, M	RMSE, PCC, SCC; AUROC, AUPR, F1 (for binarized IC50)	None
SWnet [71]	GNN + CNN	Multi (GE, Mut)	CCLE	Molecular graph	ln(IC50) GDSC and CCLE	R, CB	RMSE, R^2^	None
consDeepSignaling [18, 54]	VNN	Multi (GE, CN)	CCLE	Drug Targets	AUDRC GDSC	R	RMSE, PCC	None
HiDRA [61]	Attention-based NN	GE	GDSC	Morgan (512-bit)	ln(IC50) GDSC1	R, CB, DB	RMSE, PCC, R^2^	None
ParsVNN [53]	VNN	Mut	CCLE	Morgan (2048, r = 2)	AUDRC GDSC1 and CRTP	R, DB not applicable	PCC	None
DEEPCDR [15]	Graph CNN	Multi (GE, Mut, Meth)	CCLE	Molecular graph	ln(IC50) + Binarized IC50 GDSC1	R, leave-one-drug-out, leave-one-cell-line-out	RMSE, PCC, SCC; AUROC, AUPR (for binarized IC50)	PCC, SCC
DrugCell [41]	VNN	Mut	CCLE	Morgan (2048, r = 2)	AUDRC GDSC and CRTP	R	SCC	SCC
PathDNN [55]	VNN	GE	GDSC1	Drug Targets	AUDRC GDSC1	R, leave-one-cell-line-out	RMSE, R^2^, adjusted R^2^, PCC	None
PaccMann [17]	Attention-based NN	GE	GDSC1	Tokenized SMILES	ln(IC50) GDSC1	R, CB, DB	R^2^, RMSE	None
MOLI [16]	Auto-encoder	Multi (GE, Mut, CN)	GDSC1	None	Binarized IC50 GDSC1	N/S	AUC	None
nERD [58]	Multichannel DNN + Autoencoder	Multi (GE, CN, Mut)	CCLE & GDSC	GCN + Morgan FP	IC50	R, CB, DB	PCC, SCC, RMSE	None
MULTIDRP [59]	Hierarchical Attention (GAT + Self-Attn)	Multi (GE, CN, Mut)	CCLE	GAT + Physico-chemical	IC50	R, CB, DB	PCC, SCC, RMSE, MAE	None
GADRP [57]	Graph CNN + Autoencoder	Multi (GE, Mut, Meth, CN)	CCLE	Sparse Network (GCN)	IC50	R, CB, DB	PCC, SCC, RMSE, MAE	None

The table summarizes key characteristics of DRP models. Reference lists the model’s name and citation. Architecture specifies the model type: LLM, VNN, CNN (convolutional neural network), DNN, GNN (graph neural network), GAT. Omics describes the omics data used: GE (gene expression), Mut (mutations), Meth (methylation), CN (copy number), or Multi (multi-omics). Omics source identifies the data source. Drug specifies the drug representation method: Morgan fingerprint, SMILES, ESPF (Explainable Substructure Partition Fingerprint), FPs (Fingerprints), or drug targets. Response indicates the drug response metric: AUDRC (area under dose-response curve), AADRC (area above dose-response curve) or IC50 (Half-maximal inhibitory concentration) and the database or experimental source where that data was obtained. Split types describes the data splitting strategy: R (random), CB (cancer-blind), CTB (cancer type-blind), DB (drug-blind), B (blind) or M (missing). Overall and per drug Metrics lists performance evaluation metrics: PCC, SCC, RMSE (root mean squared error), R^2^ (coefficient of determination), ACC (accuracy), SEN (sensitivity), SPC (specificity), PRE (precision), F1 (F1-score), FPR (false positive rate), GM (geometric mean), MCC (Matthew’s correlation coefficient), AUPRC (area under the precision-recall curve), and AUROC (area under the receiver operating characteristic curve).

### Output: drug sensitivity

The choice of model output is often dictated by the nature of the available data. During drug screening experiments, data measuring cell survival or viability at various drug concentrations is collected. Some datasets provide precomputed metrics like IC50 and AUDRC, while others offer raw data, allowing for custom metric computation. When dealing with patient response data, the data typically indicates whether a patient is sensitive or resistant to a particular drug. This dichotomy requires deciding whether models should address a regression problem, predicting a continuous outcome, or a categorical one, classifying responses into distinct groups.

#### Continuous response

**Figure 4:**
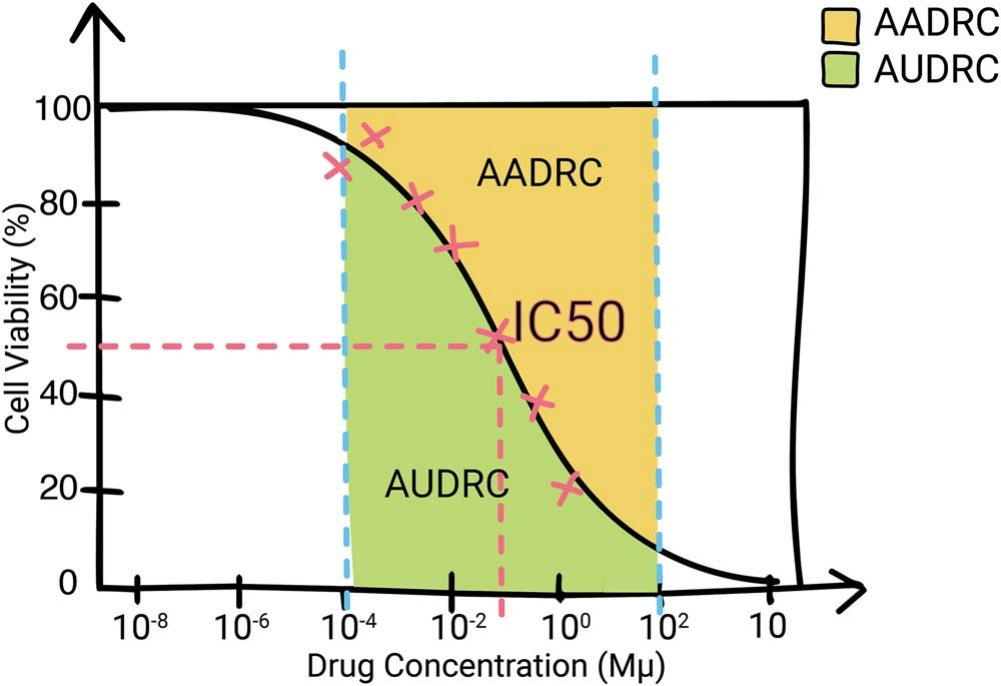
Dose–response curve with key pharmacodynamic metrics. The graph illustrates the drug’s effect across varying concentrations, marked by the IC50 value indicating the concentration at which 50% of the maximum cells are dead. The AUDRC (area under dose–response curve) quantifies the overall drug response across a predefined range of concentrations, while the AADRC (area above dose–response curve) represents the effect of the drug above the baseline response level.

As shown in the column “Response” of Table 2, for continuous response predictions, the two prevalent metrics are IC50 and AUDRC. As shown on Fig. 4, IC50 represents the drug concentration that inhibits cell growth by 50%, indicating potency but not necessarily clinical outcomes. It can be influenced by cell division rates and drug toxicity, potentially leading to the prioritization of more toxic drugs. To address these issues, alternative scores such as AUDRC and AADRC (area above dose-response curve) have been proposed. They capture the cumulative effect of the drug, are independent of dose, and better explain systematic variation in cancer drug response [31, 38].

When raw data from experiments is available, the method used to calculate metrics is very important, especially when working with different datasets for training and testing. The “Response” column in Table 2 lists the data sources used to train each model. For instance, DrugCell used GDSC and CTRP to train the model. The AUDRC was calculated using the trapezoidal rule to integrate the dose–response curve. The minimum and maximum concentrations for each experiment were set based on the specific experiment’s range [41]. However, this approach had a limitation when comparing data from different experiments that tested the same drug at varying doses. Experiments with identical outcomes for overlapping concentrations ended up with different AUDRC values simply because their maximum and minimum concentration ranges were different.

**Figure 5:**
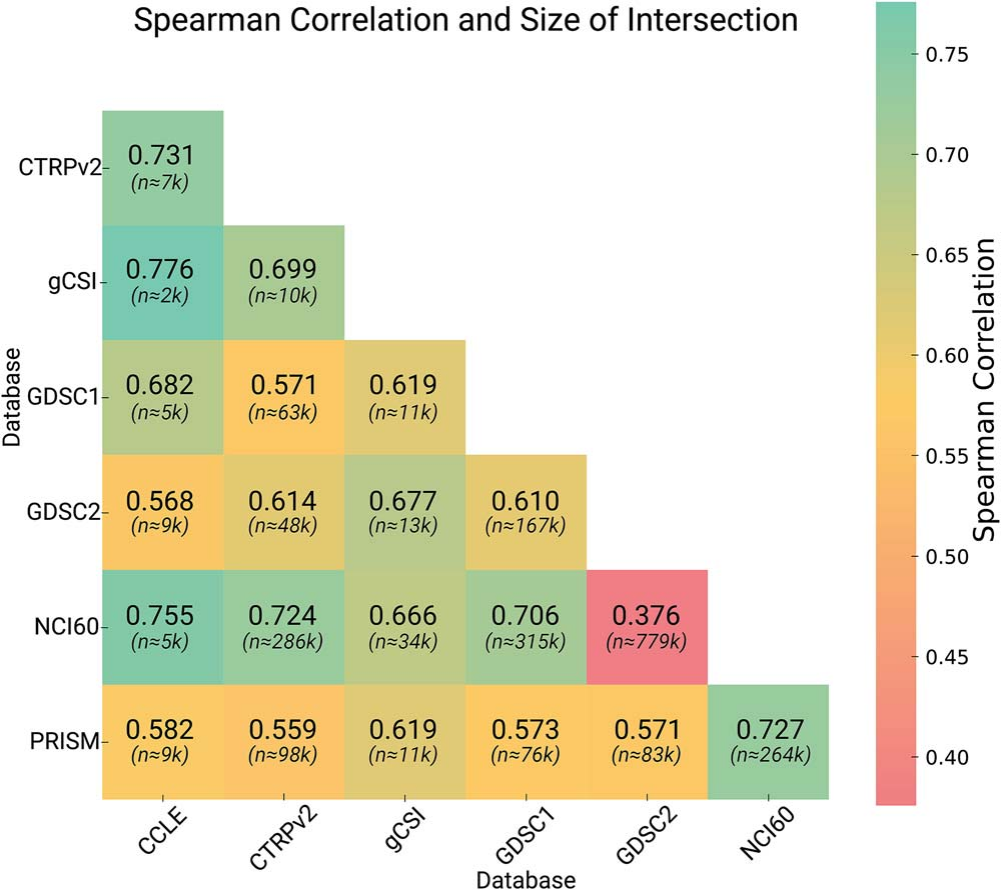
Heatmap illustrating the Spearman correlation of AUDRC values across different databases, with annotations indicating the number (n) of matching cell-drug samples. The color intensity represents the correlation strength, with reds indicating lower values and greens indicating higher values. This visualization highlights the relationships and data overlap between databases: CCLE, CTRPv2, gCSI, GDSC1, GDSC2, NCI60, and PRISM.

To overcome the inconsistencies in comparing data from different experiments, we recommend standardizing the same minimum and maximum concentrations for all drugs to, for instance,100 pM and 100 μM, respectively. Rather than relying on the trapezoidal rule, we propose using a four-parameter logistic regression model to accurately model the dose–response curve. This approach allows for precise numerical integration of the fitted curve to calculate the AUDRC. As illustrated in Fig. 5, the heatmap of Spearman correlations shows how different databases can present significantly varied drug response data, which must be considered when training with multiple databases.

After computing or obtaining the metrics, normalization techniques are usually employed. Typically, these values are scaled to fall within the range of 0 to 1, where values closer to 0 indicate a higher degree of cell death or drug effectiveness. This scaling is often done to ensure that the data is standardized, making it easier for algorithms to process and interpret the data. It helps in reducing the impact of outliers and ensures that all features contribute equally to the model training process. Normalization can also be applied per line (e.g. per patient) or per drug, depending on the specific requirements of the analysis. For instance, normalizing per drug can help in comparing the effectiveness of different drugs across various cell lines or patients. This approach ensures that the mean response for each drug is centered around zero, highlighting the differential effects of drugs on different patients. This is particularly useful in identifying drugs that have a significant impact on specific patient groups, which can be crucial for developing personalized treatment strategies.

To assess the generalizability of the models, it would be desirable to test them on a different dataset, preferably from a different institution. In a study [42], four state-of-the-art interpretable DL models were evaluated by training them on GDSC response data and subsequently testing them on CTRPv2 response data. The results revealed a notable deterioration in the performance of all models when tested on a different dataset, an outcome that is anticipated given the correlation (around 0.6) between GDSC datasets and CTRPv2 highlighted in the Spearman Heatmap we constructed (Fig. 5).

Inconsistent dose–response curve concentration ranges across experiments introduce artificial variability in IC50 calculations. When one experiment tests 0.001–10 μM and another tests 0.1–100 μM, the resulting IC50 values may not be directly comparable even for the same drug-cell line pair, as extrapolation beyond the tested range is unreliable. We suggest that standardizing to a broad range such as 100 pM - 100 μM (spanning 6 orders of magnitude) may help reduce these inconsistencies, as: (i) this range encompasses the therapeutic window of most Food and Drug Administration (FDA)-approved cancer drugs, (ii) it provides sufficient data points for robust sigmoid curve fitting (typically 8–10 concentrations), and (iii) large-scale studies [43, 44] have adopted similar standards, facilitating cross-study comparisons.

The “Independent Validation” column of Supplementary Table S1 documents the models that have undergone independent dataset testing in their original studies. For instance, PathDNN, HIDRA, and the glioma deep neural network (DNN) were trained using GDSC data and tested using CCLE data. SparseGO was trained with a dataset encompassing CCLE, GDSC1, GDSC2, and CTRPv2, and was independently validated using PRISM data. Notably, MTIGCN, MOLI, and NEST undertook a more challenging yet significantly more relevant task by using patient data from TCGA or GENIE [13] and PDX data for independent validation. As detailed in the subsequent section, they employed a binarized version of the IC50 or AUDRC to measure performance.

#### Categorical response

Drug sensitivity data can be transformed into categorical variables to represent treatment response [45]. This approach involves setting thresholds for classifying sensitivity and resistance. For example, values at or below the 25th percentile can be categorized as sensitive, while those at or above the 75th percentile can be classified as resistant. MOLI employs a binarized version of the GDSC IC50 to predict whether a cell line is a responder or non-responder [16]. MTIGCN, a multitask model, and DeepCDR, a graph convolutional neural network, predict both the IC50 value and determine whether the response will be sensitive or resistant [15, 46]. In NEST’s study, to translate predictions to discrete tumor response outcomes, they applied a threshold to the AUDRC such that predictions below a value were labeled “sensitive,” those above a value thigh were labeled “resistant” and those between these two thresholds were labeled “undefined.” For the GENIE cohort no “undefined” category was used because the number of treated samples was less than that for the earlier PDX analysis [47].

Additionally, the RECIST criteria (Response Evaluation Criteria in Solid Tumors) provide a standardized method for assessing tumor response to treatment, which is widely used in clinical settings. This involves categorizing responses into “complete response,” “partial response,” “stable disease,” and “progressive disease” based on imaging assessments. This method has been applied in studies involving genomic data, such as the evaluation of TINDL’s performance in predicting clinical drug response in cancer patients. In this context, tumor responses from the TCGA database were transformed into Boolean labels with “resistant” for stable or progressive disease and “sensitive” for complete or partial response [24].

However, using a categorical response can be controversial. While binarizing continuous data simplifies the task and makes it easier to interpret, it can also result in a loss of precision. Classifying a continuous variable into discrete categories can obscure important nuances and reduce the specificity of the predictions. Despite this, categorical responses remain a valuable tool for simplifying complex datasets and providing actionable insights.

## Algorithms

As illustrated in Fig. 1B, DRP models have evolved significantly, starting with regression methods like ridge regression, LASSO, and elastic net. These early models focused on predicting continuous values, offering accurate results but often struggling with nonlinear relationships. As the field advanced, deep learning techniques such as DNNs, CNNs, autoencoders, and attention mechanisms emerged, enabling the extraction of complex features from cells and drugs [37]. The increasing accessibility of AI resources has further driven the adoption of GNNs for processing molecular graphs, capturing intricate structural information. More recently, large language models and transformer architectures—originally developed for natural-language tasks [48]—have been adapted to therapeutic applications, leveraging self-supervised pretraining on vast corpora of sequences, text, and omics data to produce context-rich representations that enhance DRP [18–20]. All these models can be divided into two categories: specific-drug response prediction (specific-DRP) models, which focus on predicting the response to a single molecule, and general drug response prediction (general-DRP) models, which aim to predict the response across different molecules. This classification is reflected in the “Type” column of Supplementary Table S1.

A significant challenge in drug response modeling is the integration of multi-source heterogeneous data, where the feature dimensions of high-throughput omics data vastly exceed the number of available samples. Modeling strategies to manage this heterogeneity generally follow two paradigms: early integration and late (or intermediate) integration. These paradigms were proposed specifically for multi-omics clustering but can be also adopted here [49]. Early integration involves concatenating all omics data types (e.g. mutation, copy number, and expression) as input. This approach substantially increases the dimensionality of the initial input space and is prone to overfitting, making learning more difficult. Late integration addresses these limitations by combining the output of intermediate layers using different omics inputs. These independent values can be combined for the final prediction. The latter is the paradigm adopted by all the multi-source models described in this study [38]. There are multi-source methods [50–52] with the early integration approach, but they are older and it seems that the trend is using a late approach.

### Specific drug response prediction models

Specific-DRP models are designed to predict how a single drug affects cell lines based on its molecular profiles. These models employ various machine learning architectures, ranging from traditional DNNs and autoencoders to more interpretable approaches such as VNNs. For instance, NEST, a VNN-based model, constructs hierarchical knowledge representations of protein assemblies, incorporating multiple binary features per gene [13]. TINDL utilizes a dense neural network with tissue-informed normalization techniques to improve generalizability. Specifically, TINDL trains a separate model for each drug and performs a separate normalization on the GEx profiles of test samples from TCGA by calculating the gene-wise mean and standard deviation from a set of unlabeled samples corresponding to the same tissues/cancer types and then using these statistics to standardize the test samples, thereby accounting for the cancer types and tissues of origin [24]. MOLI, integrates multi-omics datasets using encoding subnetworks that are subsequently combined into a unified representation matrix before being processed by a DNN [16]. Furthermore, a glioma-specific autoencoder model has been developed to integrate omics features and uses its latent space for drug response prediction [45].

### General response prediction models

Visible neural networks, such as DrugCell and its successors, our team’s model SparseGO, and ParsVNN, utilize hierarchical representations of biological processes to predict drug sensitivity. These models combine two artificial neural networks: one processes the genotype of cell lines through a branch that represents the Gene Ontology (GO) hierarchy, and the other uses the 2048-bit Morgan fingerprint to model the drug’s chemical structure. SparseGO improves upon this framework by using a sparse neural network to represent the GO hierarchy, which significantly reduces the computational resources and time needed to train the model [32, 41]. On the other hand, ParsVNN refines DrugCell by pruning parameters to create models specific to different cancer types [53]. Other pathway-guided networks like PathDNN and consDeepSignaling integrate cell features and drug targets using connection matrices to link genes to pathways. Both models then use DNNs to process the linked information and predict drug sensitivity [54, 55].

Hybrid approaches incorporate multiple architectures to enhance predictive capabilities. DeepAEG combines a graph convolutional neural network (GCN) for chemical structure representation with a Transformer module to capture SMILES-based features [56]. A transformer-based model, DeepTTA, encodes drug features also using a Transformer to process SMILES sequences, while Quantum NN integrates a one-dimensional convolutional network for cell representation with a GCN for drug representation before feeding them into a quantum neural network layer. DeepCDR also leverages a GCN to integrate multi-omics profiles of cancer cells with drug chemical structures. It consists of a Uniform GCN for drug representation and multiple subnetworks for processing multi-omics profiles, with a 1-D CNN for final feature concatenation [15].

Similarly, GADRP [57] utilizes a graph attention network (GAT) to model the external relationships among cell lines and drugs by aggregating information from their topological neighborhoods. While NeRD [58] integrates stacked autoencoders to extract latent representations from miRNA and CNV data, MultiDRP [59] employs a hierarchical attention mechanism. Specifically, MultiDRP combines graph attention and multi-head self-attention layers to simultaneously capture the external relationships between biomedical entities and the internal correlations among specific feature items, such as genes and molecular substructures.

MMDRP uses autoencoders for cell feature extraction, a GNN for drug representation, and a fusion method to integrate multimodal input. XGDP applies GNNs to extract molecular graph features and CNNs to process gene expression data, integrating them with a multi-head attention mechanism. SWnet and HIDRA combine CNNs, GNNs, and attention-based architectures. SWnet applies a GNN for drug representation and a self-attention gene weight layer, while HIDRA includes a hierarchical artificial neural network (ANN) with networks for drug, gene-level, pathway-level, and response prediction.

### Foundation models for drug response prediction

Building on these advances, foundation-model paradigms are now being grafted onto classical pipelines. Foundation models represent an emerging paradigm in DRP, leveraging pretrained representations from large-scale biological data. These models offer several advantages for drug response prediction, particularly in scenarios with limited training data.

DeepCDR can be augmented with embeddings from scFoundation, an asymmetric transformer pretrained on >50 million single-cell transcriptomes; the resulting 768-dimensional cell representations replace the original omics branch after a 100-dimensional projection, improving IC50 prediction without additional data modalities [18]. Complementarily, TxGemma—Google DeepMind’s suite of decoder-only Gemma-2 transformers (2B, 9B, and 27B parameters) fine-tuned on ~7 million therapeutic examples—accepts free-text prompts containing a drug’s SMILES and a cell-line identifier to regress or classify drug response, toxicity, and trial outcomes, leveraging interleaved local–global attention and grouped-query attention inherited from Gemma-2 [19].

Pretrained embeddings from foundation models like scFoundation, trained on millions of single-cell RNA-seq profiles, capture fundamental patterns of cellular state and can be transferred to DRP tasks with relatively small datasets. This contrasts with traditional architectures that require substantial drug-specific training data to achieve comparable performance.

The trade-offs between foundation models and task-specific architectures with biological priors are notable. While VNNs like SparseGO incorporate explicit pathway knowledge, foundation models learn implicit biological representations from data. This data-driven approach offers flexibility but may lack the mechanistic interpretability that pathway-based models provide.

Zero-shot and few-shot learning capabilities represent a key advantage of foundation models. Models like TxGemma can generalize to entirely unseen drugs without retraining, encoding molecular structures and cellular contexts through natural language representations. This enables rapid prediction for novel compounds, though at the cost of the high-resolution biological detail captured by multi-omics architectures.

The interpretability challenges introduced by foundation models compared to pathway-based architectures remain significant. While biological priors enable clear mechanistic explanations, foundation models operate through learned representations that may not directly correspond to known biological processes. Recent advances in mechanistic interpretability offer promise, but practical clinical deployment requires methods to extract actionable explanations from these complex models.

Regardless of the chosen model, hyperparameter tuning is necessary for performance optimization. Experiment tracking and MLOps platforms like Weights and Biases [60] facilitate model development by organizing documentation and streamlining result sharing. To ensure reproducibility, final hyperparameters should be explicitly recorded.

## Validation

### Cross-validation techniques

Cross-validation is a fundamental part of developing reliable DRP models. It ensures that models are not only accurate on the data they were trained on but also when faced with new data. This is especially important in the fields of precision oncology and drug discovery, where models are used to predict outcomes for patients or drugs that were not included in the initial training dataset. Here, we explain the K-fold cross-validation strategy, though other approaches like leave-one-out or stratified K-fold cross-validation can also be used.

In K-fold CV, the data is divided into N equally sized folds. Each fold serves as the test set once, while the remaining N-1 folds are used for training. This approach ensures that every data point is used both for training and testing. This is particularly relevant because drug response can vary significantly across different drugs or cell lines. For example, a model might perform well with one drug as the test set but poorly with another. As shown in Supplementary Table S1, most studies rely on five-fold cross-validation; however, other values for N could be adopted as well.

**Figure 6:**
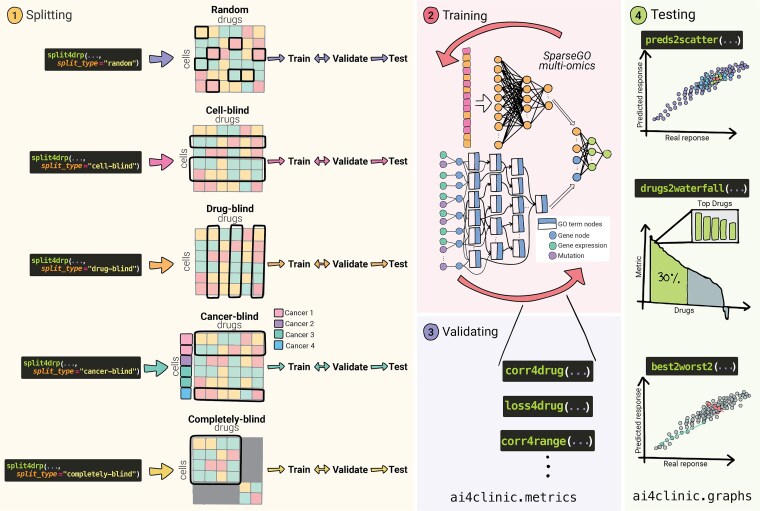
Overview of the DRP framework using the ai4clinic functions. (1) Data should be split using at least one approach other than random splitting. (2) For each splitting strategy, a model is trained and validated. As an example, the SparseGO multi-omics model is displayed. (3) During training and validation, model performance should be assessed overall and for each drug individually (it can be performed using the package). (4) Finally, the model is tested, and various graphics are generated to evaluate performance both overall and per drug.

We have outlined five cross-validation splitting approaches, displayed in Fig. 6, each tailored to address specific challenges for clinical or industrial applicability:

1. Random splitting: also known as mixed-set, leave-pairs-out or standard, this method randomly divides drug-cells pairs into K groups. As shown in Table 2, it is widely used and often performs best due to feature overlap between training and testing sets. This overlap creates an “optimistic bias,” making it a useful baseline of the model’s predictive power that serves as a benchmark against which other CV strategies can be compared. While it provides a solid starting point, it might not entirely reflect the model’s capacity to generalize to new, unseen scenarios [25, 42, 46, 61]. However, this approach is especially effective for model interpretability. When the goal is to gain a comprehensive understanding of the model’s decision-making process, its interpretability is most effectively evaluated when the model is trained using the best parameters.
2. Cell-blind: in this approach, sometimes referred to as unseen cancer or leave-cell-lines-out, cells are split into K groups; the model is trained with some cells and tested in cells excluded from the training set. This scenario is designed to simulate conditions where the model extrapolates data to untested cells, making it particularly valuable for drug repositioning and, more importantly, for precision medicine. This task is particularly significant for clinicians, as it indicates the model’s potential to guide personalized treatment selection for patients. [42, 46, 61].
3. Drug-blind: this splitting strategy, also known as unseen drugs or leave-drugs-out, drugs are divided into K groups. It involves training the model on all but a subset of drugs, which are then used for testing. It is useful when the model is intended to predict the effects of new or untested drugs on known cells, it is ideal for in-silico drug sensitivity testing. This scenario is more challenging due to the diverse response patterns and chemical characteristics of different compounds [42, 46, 61].
4. Completely-blind: the completely-blind or disjoint-set approach ensures that each fold is unique, containing no overlap between cell and drugs in the training and testing sets. This method is particularly hard because it requires the model to predict outcomes based solely on the patterns it has learned, without any prior knowledge of the tested features.
5. Cancer type-blind: the cancer-type blind splitting strategy is intended to capture relative differences between cancer subtypes. It involves grouping all cells of the same cancer type in the same splitting group. This strategy is useful for identifying potential new uses for existing drugs, as it can help to uncover if a drug that is effective for one cancer type might also be effective for another [31].

As shown on Table 2, it is surprising that most studies rely on the random splitting method, with few employing other strategies. To establish translational value, models must be assessed under at least one additional cross-validation regime that systematically withholds entire cell lines (cell-blind), entire drugs (drug-blind), or both (completely-blind). In the final section, we demonstrate that cell-blind and drug-blind strategies elicit marked performance degradation relative to random partitioning. The completely-blind setting imposes an even more stringent constraint, consistent with findings reported elsewhere [38, 42, 61].

### Evaluation metrics

The performance of models is frequently benchmarked against state-of-the-art baseline models. This comparison is required for assessing how new models perform relative to existing ones and for pinpointing areas for improvement. However, achieving accurate comparisons can be challenging due to variations in scoring metrics across different studies, often necessitating the re-training of models [25].

#### Overall and per-drug metrics

The performance of models is frequently benchmarked against state-of-the-art baseline models. This comparison is required for assessing how new models perform relative to existing ones and for pinpointing areas for improvement. However, achieving accurate comparisons can be challenging due to variations in scoring metrics across different studies, often necessitating the re-training of models [25].

The performance of DRP models should be evaluated both overall and on a per-drug basis. This dual approach allows for a comprehensive assessment of the model’s predictive power across the entire dataset while also providing detailed insights into its performance for individual drugs. The “overall metrics” and “metrics per drug” columns of Table 2 show the metrics measured by each model in both cases. As shown, many models do not measure performance for each drug individually, which limits the depth of their evaluation. As depicted in Fig. 2A, a model might exhibit high overall performance yet underperform when measuring drugs individually. This discrepancy can arise because some drugs are highly toxic, leading models to recommend them as the best option for all patients. However, these drugs will probably also be harmful to healthy tissues. Therefore, the performance of models trained on multiple drugs should always be evaluated on a per-drug basis.

Commonly used metrics for regression- and classification-based DRP models include correlation coefficients, error-based measures, and area-based scores. A description of some metrics is provided in the Supplementary Methods.

## Model interpretability

Interpretability is crucial for trust in AI, especially in healthcare, where clinicians must understand and rely on model predictions [1]. It helps identify key patient subgroups, clarify mechanisms of action and side effects, and supports transparency and regulatory compliance [34]. Yet, interpretability is not a one-size-fits-all concept—experts may understand model logic intuitively, while others need additional tools or explanations. This challenge is amplified in deep learning, where models often behave as “black boxes.”

As shown in Supplementary Table S1 (“Interpretability”), current DRP models employ different strategies such as integrating biological priors, *post-hoc* analyses, or explicit interpretability modules. Our model, SparseGO, embeds the GO hierarchy directly into its network, enabling explainable AI methods to pinpoint influential biological terms driving predictions—offering insight into both drug response and mechanisms of action.

TxGemma-Chat, the conversational variant of TxGemma, adds an interactive layer of interpretability. Given a drug’s SMILES and a cell-line ID, it outputs not only a prediction but also a natural-language rationale often grounded in molecular structure. This feature enables both global interpretability (via aggregated explanations) and patient-specific reasoning (via single-instance justifications), making AI decisions more accessible to clinicians without machine-learning expertise [19].

## Benchmarking drug-response prediction architectures

To establish a rigorous, clinically oriented assessment, we evaluated four state-of-the-art architectures—SparseGO, DeepCDR, DeepCDR+ scFoundation, and TxGemma-2B—under GDSC1 data and same protocol conditions orchestrated by our ai4clinic Python package. This toolkit automates compliant data partitioning for random, cell-blind, and drug-blind cross-validation, computes per-drug performance metrics, and generates visual summaries, thereby enforcing the best-practice standards discussed above.

### Materials

All models were trained on drug-response measurements obtained from GDSC1 (IC50 values, natural-log transformed). Cell-line features were taken from the CCLE RNA-seq matrix, normalized to TPM and log-transformed. To ensure fair comparison, we adopted the exact filtering pipeline that accompanied the original DeepCDR release: drugs lacking a PubChem identifier were excluded, and cell lines missing any omics modality were removed. After curation, the dataset comprised 107 446 records spanning 561 cell lines and 238 drugs; 19.5% of the possible 561 × 238 interaction pairs remained unmeasured. For SparseGO, DeepCDR, and DeepCDR+scFoundation, only the RNA-seq matrix was used as cell-lines features. TxGemma, in contrast, requires no omics vectors; instead, it ingests the plain cell-line name and a brief textual descriptor, a design choice that deliberately raises the difficulty relative to feature-rich competitors. Consequently, the TxGemma training set retained marginally more samples because the stringent omics completeness filter could be relaxed.

We selected these four architectures as representatives of distinct modeling paradigms in DRP. SparseGO exemplifies VNNs that integrate biological pathway knowledge, providing interpretable predictions through sparse, structured connections aligned with gene ontology. DeepCDR represents the established hybrid GNN-CNN architecture that combines graph-based drug representation with convolutional feature extraction from multi-omics data, serving as a strong baseline for multimodal integration. DeepCDR+scFoundation augments DeepCDR with embeddings from scFoundation, a single-cell RNA-seq foundation model pretrained on millions of cells, demonstrating how pretrained biological representations can enhance traditional architectures. Finally, TxGemma represents recent multimodal language models for biology, encoding both drugs and cell lines as text descriptions and leveraging the zero-shot reasoning capabilities of large language models. Together, these models span the spectrum from interpretable, knowledge-driven approaches to data-driven foundation models.

Model implementation followed these key principles:

- TxGemma prompting: we used natural language descriptions for cell lines (including cancer type and key genomic features) and drugs (SMILES-based molecular descriptions) to enable the language model to predict IC50 values.
- Hyperparameter optimization: SparseGO and DeepCDR were instantiated with the default hyper-parameters of the previous works. TxGemma-2B was fine-tuned using with LoRA (rank 8) applied to every linear projection within the query, key, value, output, gate, up, and down modules. Additional details can be found in the additional material.
- Missing data handling: there is only missing data in the response to drugs (i.e. not all the drugs are tested on all the cell-lines). The multiomics input presented no missing values. We did not impute the missing values of the response to drugs. These missing data was simply not included in the training process.

Complete implementation details, including exact hyperparameter configurations and data preprocessing pipelines, are provided in the Supplementary Methods and in our GitHub repository.

**Figure 7:**
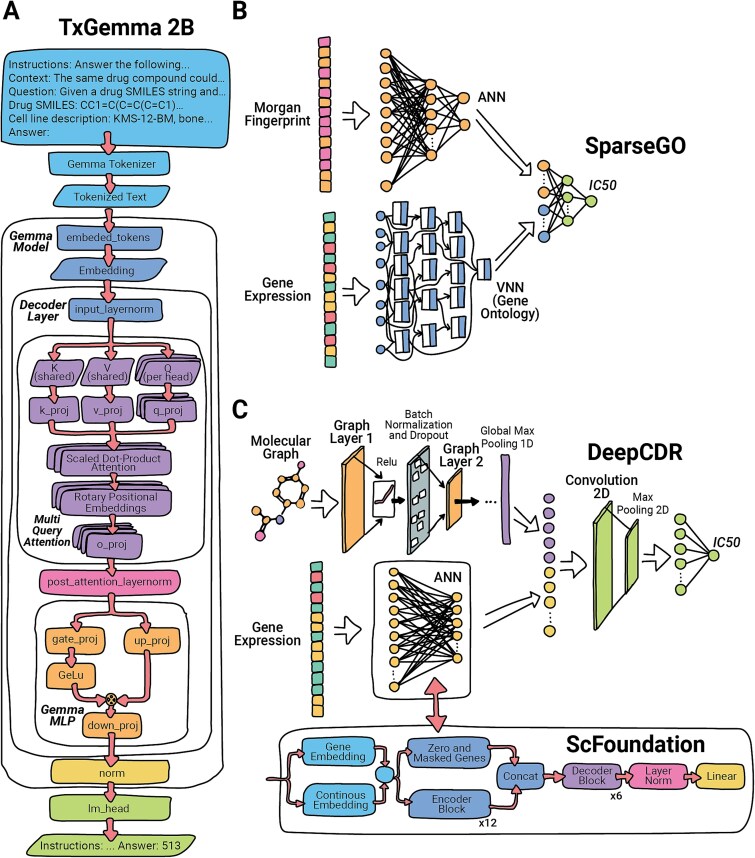
Architecture of benchmark models for general DRP. (A) TxGemma: a decoder-only Gemma-2 transformer with 2 billion parameters, designed to predict drug response by utilizing instructions, drug SMILES, and cell line descriptions through multi-query attention. (B) SparseGO: employs a sparse VNN based on Gene Ontology to encode cell-line data, combined with an ANN for drug fingerprints, to predict normalized dose–response. (C) DeepCDR: features a hybrid GCN that integrates drug molecular graphs with cell-line features using a graph convolutional branch and fully connected subnetworks, which are combined via 2D convolution and max pooling to predict IC50. DeepCDR + scFoundation enhances this framework by replacing the ANN with 768-dimensional embeddings from the transformer-based scFoundation model, pretrained on over 50 million single-cell profiles, providing richer cell representations.

Figure 7 illustrates the distinct model architectures alongside the corresponding input types used in this review. Full implementation details and optimization parameters are provided in the Supplementary Methods.

### Evaluation and results

Table 3 provides a comprehensive summary of the five-fold cross-validation results under various data splitting strategies. For each scenario, we report the average global Spearman correlation coefficient (SCC) and Pearson correlation coefficient (PCC) across all test cell-line–drug pairs, in addition to the per-drug SCC.

**Table 3:** Average performance metrics of models using different CV strategies.

Model	Split type	Average SCC	Average PCC	P/Drug SCC
SparseGO	Random	**0.87** ± 0.022	**0.89** ± 0.025	**0.56** ± 0.12
TxGemma 2B		0.84 ± 0.005	0.86 ± 0.003	0.38 ± 0.14
scFoundation		0.82 ± 0.013	0.86 ± 0.008	0.45 ± 0.12
DeepCDR		0.54 ± 0.290	0.60 ± 0.285	0.22 ± 0.13
SparseGO	Cell-blind	**0.83** ± 0.007	**0.86** ± 0.005	**0.34** ± 0.10
TxGemma 2B		0.79 ± 0.007	0.83 ± 0.003	0.13 ± 0.11
scFoundation		0.71 ± 0.082	0.75 ± 0.074	0.27 ± 0.10
DeepCDR		0.50 ± 0.245	0.56 ± 0.260	0.25 ± 0.12
SparseGO	Drug-blind	0.23 ± 0.10	0.26 ± 0.13	**0.47** ± 0.14
TxGemma 2B		**0.26** ± 0.08	**0.37** ± 0.10	0.41 ± 0.13
scFoundation		0.22 ± 0.17	0.29 ± 0.18	0.45 ± 0.13
DeepCDR		0.21 ± 0.12	0.26 ± 0.11	0.22 ± 0.15

This table outlines the average performance metrics of the models, assessed through five-fold cross-validation. The metrics include average SCC and PCC, and average per drug SCC, which collectively summarize model accuracy across different cross-validation strategies—namely, random, cell-blind, and drug-blind splits. Standard deviations indicate performance variability across folds.

While global PCC and SCC provide overall model performance assessment, these metrics can be misleading. A high global correlation often reflects the model’s ability to distinguish between drugs with vastly different potency ranges (e.g. IC50 of 0.01 μM versus 10 μM) rather than accurately ranking cell line sensitivities within a single drug. In contrast, per-drug SCC evaluates the model’s capacity to correctly order cell lines by their response to a specific drug—directly paralleling the clinical challenge of identifying which patients are most likely to benefit from a particular treatment. Therefore, per-drug SCC is the most clinically relevant metric for personalized medicine applications.

#### Random split

In the random split—the most favorable evaluation setting—all models achieve their highest global SCCs. SparseGO ranks first at 0.87 ± 0.022, followed closely by TxGemma 2B at 0.84 ± 0.005, with ScFoundation at 0.82 ± 0.013 and DeepCDR trailing at 0.54 ± 0.290.

When evaluated per drug, performance differences narrow. SparseGO attains 0.56 ± 0.12, ScFoundation 0.45 ± 0.12, and TxGemma 2B records 0.38 ± 0.14—a respectable score considering it does not rely on specialized drug or cell-line embeddings. DeepCDR remains the weakest across both metrics, with a per-drug SCC of 0.22 ± 0.13.

#### Cell-blind split

Under cell-blind conditions, global SCCs drop for all models. SparseGO leads with 0.83 ± 0.007, followed by TxGemma 2B at 0.79 ± 0.007, ScFoundation at 0.71 ± 0.082, and DeepCDR at 0.50 ± 0.245.

Per-drug rankings are more sensitive to the split: SparseGO achieves 0.34 ± 0.10, ScFoundation 0.27 ± 0.10, and DeepCDR 0.25 ± 0.12. TxGemma’s per-drug SCC drops to 0.13 ± 0.11, indicating that, in this split, the model’s reliance on textual cell-line identifiers leaves less transferable information for within-drug ranking.

#### Drug-blind split

The drug-blind split poses the greatest challenge, requiring generalization to unseen drugs. Here, TxGemma 2B achieves the highest global SCC, with 0.26 ± 0.08, outperforming SparseGO (0.23 ± 0.10), ScFoundation (0.22 ± 0.17), and DeepCDR (0.21 ± 0.12).

While SparseGO (0.47 ± 0.14) and ScFoundation (0.45 ± 0.13) slightly exceed TxGemma’s per-drug SCC (0.41 ± 0.13), these differences are modest and occur alongside TxGemma’s lead in the more demanding prediction scenario.

The benchmarking results reveal distinct performance patterns across architectures. Foundation models demonstrate particular strengths in scenarios requiring generalization to unseen drugs (drug-blind split), where pretrained representations enable zero-shot prediction capabilities. However, in cell-blind scenarios with access to detailed multi-omics data, specialized architectures with explicit biological priors maintain competitive or superior performance. This suggests that the optimal model choice depends on the specific clinical deployment scenario: foundation models excel when rapid predictions for novel drugs are needed, while pathway-based models provide advantages when comprehensive biological data is available and mechanistic interpretability is prioritized.

#### SparseGO performance in detail

**Figure 8:**
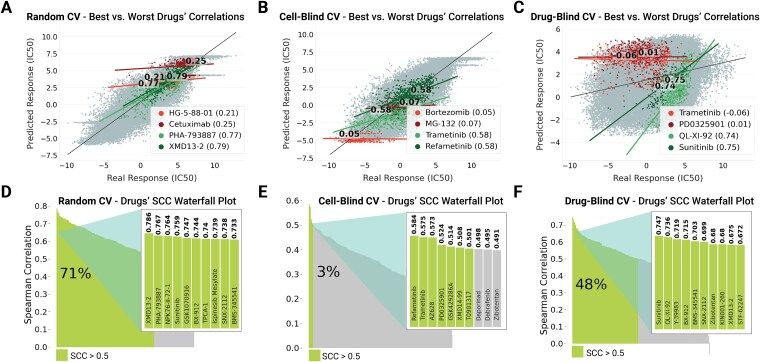
SparseGO’s performance across different cross-validation methods. This figure showcases SparseGO’s results using random (A, D), cell-blind (B, E), and drug-blind (C, F) cross-validations. Panels A–C highlight drug correlations, with red lines representing polynomial fit lines for the two drugs with the lowest correlations, and green lines indicating those with the highest correlations. The gray points represent data points for other drugs. Panels D–F display the corresponding SCC waterfall plots, emphasizing the percentage of drugs with an SCC greater than 0.5: 71% for random CV, 3% for cell-blind CV, and 48% for drug-blind CV.

Figure 8 exclusively examines SparseGO—the best-performing model—to illustrate how cross-validation choices influence drug-level accuracy. In the random-split panel (A), predicted and true log-IC_50_ values align closely for some compounds, with individual drug correlations ranging from 0.21 for HG-5-88-01 to 0.79 for XMD13–2. The accompanying waterfall plot (D) confirms that 71% of the 238 drugs exceed an SCC threshold of 0.5, indicating that SparseGO’s sparse, GO-guided architecture effectively captures robust potency orderings when both cell lines and drugs are observed during training.

When the model generalizes to entirely unseen cell lines (B), the landscape changes significantly. Bortezomib and MG-132 exhibit the weakest correlations (0.05 and − 0.07, respectively), while Trametinib and Refametinib achieve 0.58. The waterfall plot (E) shows a dramatic drop: only 3% of drugs maintain an SCC above 0.5, highlighting how the absence of familiar cellular contexts erodes ranking fidelity.

In the drug-blind scenario (C), although the global SCC falls to 0.23, individual-drug correlations are more promising. Sunitinib, QL-XI-92, Y-39983, and BX-912 surpass 0.7. Consequently, 48% of drugs retain an SCC above 0.5 (waterfall F), nearly an order of magnitude higher than the 3% observed under cell-blind conditions. This suggests that SparseGO’s structural priors allow it to preserve relative potency rankings for nearly half of the compounds, even when their chemical features are absent from the training set. Comparable visual analyses for the remaining models are provided in the Supplementary Figures.

## Discussion

In this study, we advance cancer drug response prediction by introducing a comprehensive framework for developing robust, reproducible, and clinically relevant DRP models. Our work provides a clear, step-by-step guide to model development, supported by the Python package ai4clinic, which streamlines data splitting, per-drug metric calculations, and result visualization. This contribution addresses the critical need for standardized methodologies, enhancing reproducibility and facilitating adoption in academic and clinical settings.

We focused on data quality, normalization, and diversity. We advocate for integrating multiple data sources, including omics data, to enhance model generalizability. The Spearman correlation of AUDRC values across databases—CCLE, CTRPv2, gCSI, GDSC1, GDSC2, NCI60, and PRISM—reveals significant variability in drug response data, underscoring the importance of considering database-specific characteristics when training models to ensure predictive accuracy and robustness.

Binary classification of responders versus non-responders represents an important clinical endpoint that warrants dedicated investigation. While our study focuses on regression-based IC50 prediction, classification approaches face unique challenges including threshold selection, class imbalance handling, and appropriate evaluation metrics. Future work should systematically explore these factors, as the optimal threshold for defining response may be drug specific and context dependent. This requires careful integration with clinical response criteria such as RECIST and consideration of precision-recall trade-offs for imbalanced datasets.

We advocate for rigorous validation protocols beyond conventional random splitting, which often overestimates model performance due to its limited clinical relevance. By using cell-blind and drug-blind cross-validation strategies, we ensure models generalize to unseen cell lines or compounds—a critical requirement for clinical applicability where predictions must extend to new patients and treatments. To our knowledge, this study is among the first to rigorously test TxGemma-2b using these approaches.

A limitation of our benchmarking study is the reliance on GDSC data alone. Although cross-dataset validation would strengthen claims of robustness to experimental heterogeneity, we restricted the analysis to GDSC to ensure a fair and controlled comparison with the reference foundation and deep learning models (scFoundation, DeepCDR, and TxGemma), which were originally optimized for this dataset. Importantly, this limitation pertains to the scope of the current benchmarking study rather than to the intrinsic transferability of specific architectures. For example, the SparseGO architecture has previously been evaluated across multiple pharmacogenomic datasets (GDSC, CTRP, and CCLE) in an independent study [32], whereas comparable standardized cross-dataset evaluations have not yet been reported for the other reference models. Future work should systematically investigate cross-dataset generalization using harmonized preprocessing and domain-adaptation strategies.

Although SparseGO led in most scenarios, TxGemma 2B achieved the best performance in the most challenging drug-blind split and in other settings delivered results that were remarkably close to the leader. This is particularly noteworthy given that TxGemma was not explicitly provided with any cell-line omics data—relying solely on a name and a brief description—and received the drug directly as a SMILES string, without any pre-computed molecular features or embeddings.

We hypothesize that TxGemma’s predictive performance can be significantly strengthened through several strategic enhancements. First, implementing more rigorous hyperparameter tuning using cell-blind validation can optimize performance in scenarios where standard random splits may overestimate generalizability. An approach proven effective in enhancing robustness in SparseGO. Second, adoption of novel parameter-efficient fine-tuning methods such as DoRA (weight-decomposed low-rank adaptation) could surpass the performance of standard LoRA approaches by separating updates into magnitude and directional components, enhancing both learning capacity and stability without increasing inference cost [62]. Finally, developing a next-generation model based on an improved foundation—a hypothetical “Gemma-3–based” variant—may yield stronger baseline performance. The recently released Gemma 3, a successor to Gemma 2, offers enhanced multimodal capabilities, extended context windows (up to 128K tokens), improved architectural efficiency, and superior performance in downstream tasks compared to its predecessor [63]. Building TxGemma atop such a foundation could enable more effective representation learning from sparse inputs like cell lines and drug descriptors [48].

Beyond these specific enhancements, foundation models offer distinct advantages and trade-offs compared to specialized architectures. In low-data regimes, FMs like scFoundation leverage massive pretraining (in this case, on millions of single-cell profiles) to provide robust feature representations even when drug-specific training data is sparse. This contrasts with VNN-based models like SparseGO, which rely on well-curated biological priors (pathway databases) and may struggle when these priors are incomplete or when drugs act through novel mechanisms.

Text-based representations used in TxGemma present notable limitations. While natural language descriptions offer flexibility, they inherently lack the high-resolution biological specificity provided by raw multi-omics data. For instance, describing a cell line as “lung adenocarcinoma with EGFR mutation” loses the quantitative gene expression signatures that GNN-based models exploit. Our benchmarking indicates that TxGemma’s strength lies in generalization to entirely unseen drugs (drug-blind split), where its language-based molecular understanding provides an advantage, but it underperforms when detailed omics information is available.

This reveals a fundamental interpretability trade-off. VNNs provide pathway-level explanations grounded in established biology, making predictions more actionable for clinicians. Foundation models, while powerful, often operate as black boxes despite their scale. Recent work on mechanistic interpretability of transformers —particularly attention-based analysis of pathway-level biological processes—offers promise for partially addressing this limitation. However, such mechanistic interpretability remains less clinically actionable than the explicit pathway-level explanations provided by VNNs, suggesting that the choice between these approaches depends on whether mechanistic understanding or rapid generalization to novel compounds is prioritized.

The multimodal capabilities of these foundation models offer additional opportunities for advancement. Transformer-based architectures can simultaneously process genomic, transcriptomic, proteomic, and clinical text data, enabling unified representations of cellular states and drug mechanisms. Recent work [64] has demonstrated that pathway-informed transformers combining multi-omics inputs with domain knowledge achieve superior performance in predicting drug response, cancer types, and subtypes. Moreover, interpretability can be partially addressed through mechanistic analysis of attention patterns, revealing pathway-level biological processes relevant to specific drug-cell pairs and potentially accelerating biomarker discovery. However, such mechanistic interpretability remains less clinically actionable than the explicit pathway-level explanations provided by VNNs.

### Practical implementation considerations

Successful clinical adoption requires addressing reproducibility and accessibility challenges. Comprehensive documentation, detailed environment specifications, and intuitive user interfaces are essential to overcome barriers posed by poorly documented code repositories, which often hinder independent replication and implementation. Hardware requirements vary significantly across architectures: SparseGO operates efficiently on a standard 8GB GPU, making it accessible for most research environments. TxGemma-2B also fits on standard GPUs, though LoRA training requires larger GPUs. In our study, we utilized the smallest TxGemma model to maximize accessibility. However, for TxGemma-27B, high-end GPUs (A100 or H100) are necessary, as this model cannot be accommodated on a personal computer. These practical constraints must inform model selection for resource-constrained clinical settings.

### Challenges in translating research to clinical practice

The translation of drug response research from cell lines or PDCs to patients is a complex task, primarily due to significant biological differences, experimental bias, and the scarcity of patient data. As discussed, cell lines, while useful for initial screening and testing, do not fully replicate the complexity of human biology [23]. Ideally, models should be trained with *in vivo* data, but the limited availability of patient records with detailed drug response information makes it challenging to train robust machine learning models directly with patient data [16]. Furthermore, for models to be used by oncologists, they must undergo experimental validation, a step often inaccessible to many research labs.

These experiments could include cytotoxicity assays to test novel drug repositioning and validate the interpretability of the models. For example, cytotoxicity assays can be used to assess the impact of repurposed drugs on cancer cell lines, ensuring that the predicted drug responses are accurate and reliable. Furthermore, experiments should be designed to validate that the features identified by DRP models as important are, in fact, biomarkers or drug targets. For example, a study could investigate whether the genes and pathways highlighted by a DRP model as significant are directly involved in the drug’s mechanism of action. This validation is essential for building trust in the model’s predictions and ensuring their clinical relevance. Only a few models, such as MTIGCN, TINDL, DrugCell, and MOLI, have been validated using datasets other than cell lines. Among these, TINDL, DrugCell, MOLI, HiDRA, and NEST have performed some form of experimental validation.

In addition, current models predict intrinsic sensitivity but ignore drug bioavailability, metabolism, and patient-specific dosing—critical factors for clinical efficacy. Hybrid models that combine cell line predictions with PK/PD parameters could provide more clinically actionable predictions, accounting for the complex interplay between drug exposure and therapeutic effect. On the other hand, cell lines lack the complex Tumor Microenvironment (TME) interactions (immune cells, stromal cells, and hypoxia) that modulate drug response *in vivo*. Future models should incorporate spatial transcriptomics data and TME-aware embeddings to capture these interactions. Recent advances in spatial profiling technologies offer opportunities to bridge this gap.

In conclusion, our work underscores the need for a multifaceted approach in developing and validating drug response prediction models. We emphasize the importance of data quality, rigorous validation with clinically relevant strategies, interpretability, and practical implementability. The integration of large language model (LLMs) holds significant potential, as they can process and learn from diverse biological data types, offering more nuanced and accurate predictions.

Future efforts should focus on collecting and curating larger, more diverse patient datasets and conducting thorough experimental validations. This will facilitate the successful translation of DRP models into valuable tools for precision oncology. Additionally, exploring the inherent multimodal capabilities of LLMs could unlock new avenues for integrating genomic, transcriptomic, proteomic, and clinical text data, thereby enhancing the predictive power and clinical applicability of these models.

**Key Points**

- Foundation and deep learning models are advancing cancer drug response prediction by integrating diverse omics data and improving generalization across biological contexts.
- However, the field lacks standardized data integration, validation, and clinically relevant evaluation metrics—gaps we address through a unified framework designed to enhance reproducibility and translational reliability.
- Benchmarking interpretable (SparseGO) and foundation (TxGemma) architectures demonstrates how combining biological insight with large-scale pretraining yields robust, clinically meaningful predictions.

## Supplementary Material

Supplementary-short_bbag225

## Data Availability

The raw data used to calculate the AUDRC for the CCLE, CTRPv2, gCSI, GDSC1, GDSC2, NCI60, and PRISM datasets was obtained through the PharmacoGx R [60] package. The SparseGO codebase and training data, together with the implementation used for AUDRC calculation, are available at https://github.com/KatynaSada/SparseGO_lightning. Code to reproduce the TxGemma results on GDSC is provided at https://github.com/KatynaSada/TxGemma_DRP. The code used to reproduce the scFoundation and DeepCDR results is available at https://github.com/biomap-research/scFoundation/tree/main/DeepCDR. The ai4clinic package is distributed through PyPI (https://pypi.org/project/ai4clinic) and its source code is hosted on GitHub (https://github.com/KatynaSada/ai4clinic). The repository also includes an example Jupyter notebook illustrating how to use the package.
