# Supplementary material for: Foundation models and deep learning for cancer drug response prediction: a framework for data, metrics, and validation
Source: Brief Bioinform. 2026 May 19;27(3):bbag225. doi: 10.1093/bib/bbag225 (PMC13184516; doi:10.1093/bib/bbag225)
Supplement: Supplementary-short_bbag225 [file supplementary-short_bbag225.docx]

**Supplementary: Foundation Models and Deep Learning for Cancer Drug Response Prediction: A Framework for Data, Metrics, and Validation**

# Authors

Katyna Sada Del Real^1^, Vinay S. Swamy^3^, Josefina Arcagni^1^, Eric Wang^4^, Raul Rabadan^3^, Angel Rubio^1,2*^

^1^ Departamento de Ingeniería Biomédica y Ciencias, TECNUN, Universidad de Navarra, San Sebastian, Spain.

^2^ Instituto de Ciencia de Datos e Inteligencia Artificial (DATAI), Universidad de Navarra, 31080, Pamplona, Spain

^3^ Department of Biomedical Informatics, Columbia University, USA

^4^ Google DeepMind

*Correspondence for materials and methods should be addressed to Angel Rubio (email: [arubio@unav.es](mailto:arubio@unav.es))

**Keywords**: cancer drug response, machine learning, metrics, framework

# Table of Contents

1. Supplementary Tables
 1.1 Supplementary Table 1 – Overview of DRP Models

2. Supplementary Methods
 2.1 Data Preprocessing (Omics)
  2.1.1 DNA Mutations
  2.1.2 Gene Expression
  2.1.3 Copy Number Variation (CNV)
  2.1.4 DNA Methylation
 2.2 Data Integration
 2.3 Drug Parametrization Methods
  2.3.1 Linear Notations
  2.3.2 Molecular Fingerprints & DL Embeddings
  2.3.3 Graph Structures
  2.3.4 Drug Targets
 2.4 Performance Metrics
 2.5 Architectures (Model-Specific Details)

3. Supplementary References

# 1. Supplementary Tables

## 1.1 Supplementary Table 1 – Overview of DRP Models

Supplementary Table 1. Overview of DRP Models. The table summarizes key characteristics of DRP models. MODEL lists the model names, while ARCHITECTURE specifies the model type: VNN (Visible Neural Network), CNN (Convolutional Neural Network), DNN (Deep Neural Network), GAT (graph attention network), or GNN (Graph Neural Network). TYPE indicates whether the model is trained for only one drug (specific) or several drugs (general). OMICS describes the omics data used: GE (Gene Expression), Mut (Mutations), Meth (Methylation), CN (Copy Number), or Multi (Multi -omics). OMICS SOURCE identifies the data source. DRUG specifies the drug representation method: Morgan fingerprint, SMILES, ESPF (Explainable Substructure Partition Fingerprint), FPs (Fingerprints), or drug targets. RESPONSE indicates the drug response metric: AUDRC (Area Under Drug Response Curve), AADRC (Area Above Drug Response Curve) or IC50 (Half-maximal inhibitory concentration). RESPONSE SOURCE specifies the database or experimental source of the drug response data. CV (Cross -Validation) indicates the number of folds used for model training, and INDEP. VALIDATION states whether the model was tested on an independent dataset. SPLIT TYPES describes the data splitting strategy: R (Random), CB (Cancer-Blind), CTB (Cancer Type-Blind), DB (Drug-Blind), B (Blind) or M (Missing). OVERALL AND PERDRUG METRICS lists performance evaluation metrics: PCC (Pearson Correlation Coefficient), SCC (Spearman Correlation Coefficient),RMSE (Root Mean Squared Error), R² (Coefficient of Determination), ACC (Accuracy), SEN (Sensitivity), SPC (Specificity), PRE (Precision),F1 (F1-score), FPR (False Positive Rate), GM (Geometric Mean), MCC (Matthew’s Correlation Coefficient), AUPC (Area Under the Precision-Recall Curve), AUPRC (Area Under the Precision-Recall Curve), and AUROC (Area Under the Receiver Operating Characteristic Curve).INTERPRETABILITY specifies the interpretability method used. N/S indicates cases where something is not specified.

| Reference | ArchitecTure | Type | Omics | Omics Source | Drug | Response | Response Source | CV | Independent Validation | Split types | OVERALL METRICS | METRICS PER DRUG | Interpretability |
| --- | --- | --- | --- | --- | --- | --- | --- | --- | --- | --- | --- | --- | --- |
| TX-GEMMA [1] | LLM | General-DRP | Cell line name | GDSC1 and GDSC2 | SMILE | ln(IC50) | GDSC1 and GDSC2 | N/S | No | R | PCC | None | TxGemma-Chat |
| Scfoundation | Transformer/  LLM | General-DRP | GE | GDSC2 | MolecularGraph | ln(IC50) | GDSC2 | N/S | No | R | PCC | None | None |
| Prophet | Transformer | General-DRP | Multi | CCLE | Fingerprint | Multiple | Multiple | N/S | Yes | R, CB, DB | PCC | Partial | None |
| XGDP [2] | Graph CNN | General-DRP | GE | CCLE | GNN | IC50 | GDSC | 3-fold | No | R, DB | RMSE, PCC, R² | None | GNNExplainer |
| MMDRP [3] | GNN | General-DRP | Multi, GE, Mut, CN… | CCLE | Molecular graph | AADRC | CTRPv2 | 5-fold | No | CB, DB, CTB, B | MAE, RMSE | None | Integrated Gradients |
| nest [4] | VNN | Specific-DRP | Mut, CN | CCLE | None | AUDRC + Binarized AUDRC | CTRP and GDSC | 5-fold | PDX and GENIE | DB not applicable | - | PCC, diagnostic odds ratio | RLIPP |
| DeePAEG [5] | Graph CNN | General-DRP | Multi (GE, Mut, Meth, CN) | CCLE | ESPF of SMILES | ln(IC50) | GDSC | N/S | No | R | PCC, SCC, RMSE (and per cancer type) | PCC, SCC, RMSE | Multivariate function |
| Glioma DNN | DNN | Specific-DRP | Multi (GE, Mut) | GDSC | None | IC50 + Binarized IC50 | GDSC | 10-fold | CCLE | R, DB not applicable | - | ACC, SEN, SPC, PRE, F1, FPR, GM, MCC | None |
| HQNN [6] | Quantum NN | General-DRP | Mut | GDSC | Molecular graph | IC50 | GDSC | No | No | R | RMSE | None | None |
|  |  |  |  |  |  |  |  |  |  |  |  |  |  |
| MTIGCN | Graph CNN | General-DRP | GE | GDSC and CCLE | DL embedding of PubChem FP | IC50 + Binarized IC50 | GDSC and CCLE | N/S | PDX and TCGA | R, CB, DB, M | PCC, SCC, RMSE; AUPC, AUPRC (for binary task) | None | None |
| TINDL [7] | DNN | Specific-DRP | GE | GDSC | None | ln(IC50) | GDSC | 5-fold | TCGA | R, DB not applicable | - | PCC, P-value (for TCGA) | Averaged CXPlain scores |
| SparseGO [8] | VNN | General-DRP | Mut or GE | GDSC1, GDSC2, CTRPv2 and CCLE | Morgan (2048, r=2) | AUDRC | GDSC1, GDSC2 and CTRPv2 | 5-fold | PRISM | R, CB, DB | PCC, SCC, RMSE | PCC, SCC | DeepLIFT + SVMs |
| DeepTTA [9] | Transformer + NNs | General-DRP | GE | GDSC2 | ESPF of SMILES | ln(IC50) | GDSC2 | No | No | R, CB, DB, CTB, M | RMSE, PCC, SCC; AUROC, AUPR, F1 (for binarized IC50) | None | None |
| SWnet [10] | GNN + CNN | General-DRP | Multi (GE, Mut) | CCLE | Molecular graph | ln(IC50) | GDSC and CCLE | No | No | R, CB | RMSE, R² | None | Weights layer |
| consDeepSignaling | VNN | General-DRP | Multi (GE, CN) | CCLE | Drug Targets | AUDRC | GDSC | 5-fold | No | R | RMSE, PCC | None | SmoothGrad |
| HiDRA [11] | Attention-based NN | General-DRP | GE | GDSC | Morgan (512-bit) | ln(IC50) | GDSC1 | 5-fold | GDSC1, GDSC2, CCLE | R, CB, DB | RMSE, PCC, R² | None | Hierarchical attention |
| ParsVNN [12] | VNN | Cancer-specific General-DRP | Mut | CCLE | Morgan (2048, r=2) | AUDRC | GDSC, CRTP | 5-fold | No | R, DB not applicable | PCC | None | Pruning |
| DEEPCDR [13] | Graph CNN | General-DRP | Multi (GE, Mut, Meth) | CCLE | Molecular graph | ln(IC50) + Binarized IC50 | GDSC | 5-fold | TCGA | R, CB, DB | RMSE, PCC, SCC; AUROC, AUPR (for binarized IC50) | PCC, SCC | Scores to genes based on the absolute gradient of the predicted outcome. |
| DrugCell [14] | VNN | General-DRP | Mut | CCLE | Morgan (2048, r=2) | AUDRC | GDSC and CRTP | 5-fold | PDX Encyclopedia | R | SCC | SCC | RLIPP |
| PathDNN  [15] | VNN | General-DRP | GE | GDSC | Drug Targets | AUDRC | GDSC | 10-fold | CCLE | R, leave-one-cell-line-out | RMSE, R², adjusted R², PCC | None | Sparse layer of pathway nodes |
| PaccMann [16] | Attention-based NN | General-DRP | GE | GDSC | Tokenized SMILES | ln(IC50) | GDSC | 25-fold | No | R, CB, DB | R², RMSE | None | Gene attention values |
| MOLI [17] | Auto-encoder | Specific-DRP | Multi (GE, Mut, CN) | GDSC | None | Binarized IC50 | GDSC | 5-fold | PDX Encyclopedia and TCGA | N/S | AUC | None | None |
| NERD | Multichannel DNN + Autoencoder |  | Multi (GE, CN, Mut) | CCLE | GCN + Morgan FP | ln(IC50) | PRISM | 5-fold | None | R, CB, DB | RMSE, SCC, PCC, R² | None | Shapley values |
| MULTIDRP | Hierarchical Attention (GAT + Self-Attn) |  | Multi (GE, CN, Mut) | CCLE | GAT + Physico-chemical | log(IC50) | PRISM | 80/10/10 split plus averaging of 10. | GDSC, TCGA | R, CB | RMSE, SCC, PCC, R² | None | Pathway association, GO Biological process enrichment |
| GADRP | Graph CNN + Autoencoder |  | Multi (GE, CN, Meth, Mut) | CCLE | Sparse Network (GCN) | log(IC50) | PRISM | 5-fold | None | R | RMSE, SCC, PCC, R² | None | GO enrichment, Network proximity |

# 2. Supplementary Methods

## 2.1 Data Preprocessing (Omics)

The preprocessing of the different omic data types is essential to ensure the data is in a suitable format for machine learning analysis. DNA mutations are identified using technologies such as Illumina HiSeq [18–20] sequencing platform. The process of mutation detection involves sequencing the samples and comparing the sequences to a reference genome to identify deviations. Once the mutations are identified, the mutation data undergoes a binarization process to indicate the presence (1) or absence (0) of specific mutations within the cells. This step is required for transforming the mutation data into a format that can be easily integrated into machine learning models as features. The decision of which types of mutations to include in the analysis is also important. Commonly, all non-synonymous coding mutations are included, as these are more likely to affect protein function. However, depending on the specific research question or the complexity desired in the model, a stricter or looser criterion can be applied to select only the relevant mutations [21].

Gene expression data is obtained from either arrays or RNAseq, as in CCLE and GDSC. The initial step in processing this data involves normalizing raw read counts to account for differences in sequencing depth and gene length. For microarray data (e.g. Affymetrix), normalization often uses the Robust Multi-array Average (RMA) method, which corrects for background noise and array variations [15]. For RNA sequencing data (e.g. Illumina), metrics such as Fragments Per Kilobase of transcript per Million mapped reads (FPKM) or Transcripts Per Million (TPM) are used to normalize read counts by gene length and total reads. After normalization, a log2(FPKM + 1) transformation is frequently applied. Other normalization methods, such as Trimmed Mean of M-values (TMM) normalization or upper quartile scaling, may be implemented to adjust raw counts and ensure comparability of expression values across samples. Additionally, either using mutations or expression, genes can be filtered to focus on the most informative features for machine learning models. For instance, genes exhibiting low variability across different cell lines can be excluded [17,22,23]. Other feature selection methods include choosing genes associated with known pathways, identifying genes that show a high correlation with responses to specific drugs in the training set or using the genes from the LINCS-1000 project—as these genes capture a significant amount of cell status [24].

CNV data is acquired through platforms such as Agilent, NimbleGen, Affymetrix, and Illumina, which are used in datasets like NCI60 and CCLE. The data undergoes normalization to address technical biases that can arise from the sequencing or array data. Following normalization, segmentation is performed to pinpoint the exact regions of the genome that have experienced gains or losses in copy number. After segmentation, the CNV data is summarized and quantized, discretizing it into clear categories like copy-neutral, amplifications, or deletions. These discretized data segments are then used as features in machine learning models.

DNA methylation data, available in datasets such as NCI60, CCLE, and GDSC I, is derived from platforms such as the Infinium HumanMethylation450k BeadChip array, Illumina Human Methylation 450 Array or bisulfite sequencing. The preprocessing of methylation data includes normalization to correct for biases introduced during the bisulfite conversion process and to adjust for differences in sample preparation and measurement. Following normalization, a beta-value transformation is applied, representing methylation levels as a proportion of the total combined methylated and unmethylated signal, providing a scale between 0 and 1. This transformation is required for its integration as features of ML models.

## 2.2 Data Integration

As discussed, omics data from sources like CCLE and GDSC are often generated using different platforms, leading to variations in data magnitude and distribution. When combined for model training, these discrepancies introduce biases that can impact performance. To mitigate these issues, normalization techniques such as z-score normalization and quantile normalization are applied to standardize data scales. Additionally, batch correction methods like ComBat help to align datasets by reducing batch effects, improving overall model reliability. However, a limitation of ComBat is that it adjusts gene expression profiles as a preprocessing step, requiring retraining when predicting drug responses for new cancer patients [7,24,25].

Beyond normalization, preprocessing steps ensure consistency and comparability across datasets. In the development of the model MOLI, gene expression values were standardized by obtaining raw intensities from ArrayExpress for the GDSC dataset, which were then RMA-normalized, log-transformed, and aggregated to the gene level. For PDX and TCGA datasets, gene expression values were converted to TPM and log-transformed. FPKM values for PDX samples were also converted to TPM and log-transformed. Gene expression was standardized and a pairwise homogenization procedure was performed to ensure comparability across different platforms. The 5% of genes with the lowest variance were excluded as they were deemed not informative [17].

For somatic copy number profiles, preprocessing involved removing unreliable segments from genome segmentation files for TCGA datasets and assigning every gene a value corresponding to the intensity log-ratio of the segment it overlaps. In MOLI, if a gene overlaps more than one segment, the most extreme log-ratio value was kept. Alternatively, one could consider the average copy number for the gene or even evaluate genes with copy number changes individually. [26] For GDSC and PDX datasets, the logarithm of the copy number divided by the sample's ploidy in the copy-neutral state was computed for every gene. Finally, gene-level copy number estimates were binarized [17].

These preprocessing techniques play a crucial role in reducing discrepancies between datasets, ensuring more accurate model training, and improving the translatability of predictive models for clinical applications.

## 2.3 Drug Parametrization Methods

2.3.1 Linear Notations

Linear notations encode molecules as strings, with SMILES (Simplified Molecular-Input Line-Entry System) and InChI (International Chemical Identifier) being the most common. SMILES strings, composed of ASCII characters, represent chemical structures in a human-readable format, capturing both 2D and 3D molecular information [27]. SELFIES (SELF-referencIng Embedded Strings) is a newer method for representing molecules computationally. Unlike SMILES, which can produce invalid strings, SELFIES ensures that every string corresponds to a valid molecule, offering a robust alternative worth exploring [28]. These strings are introduced into models through encoding methods like one-hot encoding or tokenization, which break down the linear notation into substructures for processing by neural networks or LLMs. For example, the PaccMann model employs an attention-based encoder to process tokenized SMILES [16].

2.3.2 Molecular Fingerprints and Deep Learning Embeddings

Molecular fingerprints are fixed-length bit strings that encode the presence or absence of molecular substructures. They are advantageous due to their simple mathematical structure, making them suitable for machine learning tasks. Structural keys, such as MACCS Keys and Chemically Advanced Template Search, use binary strings to encode functional groups. However, they lack information on the relative positions of these groups. Circular fingerprints, like Extended Connectivity FPs (ECFPs) based on the Morgan algorithm, address this by iteratively searching substructures. Morgan Fingerprints, a type of ECFP, are generated by enumerating substructures within a specified radius around each atom. PubChem Fingerprints are another common type, encoding chemical structures as binary bit-strings. Despite their simplicity, fingerprints are powerful in many applications, though they may not capture 3D structural information [27]. Notably, one of the most well-known DRP models, DrugCell, uses the 2048-bit Morgan fingerprint as input to a VNN, an approach that has been adopted by other models as well.

The Explainable Substructure Partition Fingerprint (ESPF), employed in DeepTTA and DeepAEG, offers a novel approach by decomposing drugs into moderate-sized substructures with strong predictive values [9]. Inspired by natural language processing techniques, ESPF uses the Byte Pair Encoding algorithm to identify frequent subsequences in databases [5,29].

Beyond traditional fingerprints, deep learning has introduced new methods for creating molecular embeddings. Autoencoders, including standard autoencoders, graph autoencoders, convolutional neural network (CNN) autoencoders, and variational autoencoders, can be trained to recreate molecular representations. The latent space of these autoencoders, which has less dimensions and is more informative, can serve as an input for a model. [30] Regular CNNs can also be used to generate embeddings. For instance, MTIGCN employs a neighborhood interaction-based heterogeneous graph CNN to learn feature representations of cell lines and drugs separately and then utilizes the generated embeddings as inputs to the DRP model [31].

2.3.3 Graph Structures

Graph notations provide a more detailed representation by encoding molecules as graphs, where atoms are nodes and bonds are edges [2]. This approach captures structural and chemical properties. SWnet leverages this approach by using a graph neural network (GNN) to process the molecular graphs and a CNN for gene features, incorporating multi-tasking and self-attention functions to assess compound similarity [10]. Chemoinformatics toolkits, such as RDKit, offer libraries for generating fingerprints and transforming SMILES into molecular graphs.

2.3.4 Drug Targets

Databases like DrugBank and STITCH provide annotations about drugs and their targets, which can be used as features in models. These annotations may be binarized or assigned confidence scores, as seen in PathDNN, where STITCH confidence scores are used as input [15].

The effectiveness of drug molecular representations varies across different ML models and DRP tasks. Exploring different inputs and methods for feature selection and dimensionality reduction is recommended.

## 2.4 Performance Metrics

The key metrics that are commonly employed to measure performance of regression-based DRP models. These metrics should be calculated not only across the entire test dataset but also separately for each drug and, if possible, within the critical range where the drug demonstrates higher sensitivity.

1. Pearson and Spearman Correlation Coefficient and R-squared (R²): These metrics measure the relationship between observed and predicted drug response measures. They provide a quantitative assessment of how well the model's predictions align with actual outcomes.
2. Mean Squared Error (MSE): This metric evaluates the average squared difference between observed and predicted values. It offers a clear indication of the model's accuracy, with lower values indicating better performance.

For classification tasks within DRP models, the following metrics are also relevant:

1. Area Under the Curve (AUC): The AUC represents the entire two-dimensional area under the Receiver Operating Characteristic (ROC) curve, which plots the true positive rate against the false positive rate at various threshold settings. AUC is a widely used metric for binary classification tasks, with values closer to 1 indicating better model performance.
2. Area Under the Precision-Recall Curve (AUPRC): The AUPRC is another performance metric, specifically tailored for imbalanced datasets, where one class is much more prevalent than the other. It measures the average precision and recall across different classification thresholds, providing a more nuanced view of the model's performance in classification tasks.

## 2.5 Architectures (Model-Specific Details)

SparseGO was instantiated with its default hyper-parameters optimized in prior work: a sparse visible neural network of 3 065 Gene Ontology terms (max depth 8; ≥5 annotated genes per term; ≥30 distinguishing genes from children) feeds six neurons per GO term and 24 neurons in the final GO layer. Drug structures are encoded by an auxiliary ANN (200-100-50 neurons) acting on 2048-bit Morgan fingerprints. The two latent vectors are concatenated and passed through a 20-neuron fully connected layer. Training proceeded with SGD (learning rate 0.01, momentum 0.93), MSE loss, batch size 15 000, and dropout rates of 0.15 on genes, 0.05 on GO terms, and zero elsewhere.

DeepCDR was retrained as published: a uniform graph convolutional network processes molecular graphs while a separate subnetwork digest RNA-seq; features are fused by a 2-D CNN and regressed to IC50. DeepCDR+scFoundation replaced the RNA-seq branch with 768-dimensional embeddings produced by the foundation model, followed by a 100-neuron linear projection before concatenation with drug and other omics embeddings. Both variants used the original hyper-parameter grid and early-stopping protocol provided by the authors.

TxGemma-2B was fine-tuned with LoRA (rank 8) applied to every linear projection within the query, key, value, output, gate, up, and down modules. We froze the full 2 B-parameter backbone and updated only the low-rank adapters, training with AdamW at a learning rate of 5 × 10⁻⁶, a micro-batch size of 10 across four GPUs, and a maximum sequence length of 512 tokens. All predictions were generated via zero-shot prompting—that is, prompts contained no examples or demonstrations whatsoever, forcing the model to rely solely on its pretrained and adapter-acquired knowledge. Whereas the original TxGemma study evaluated exclusively under random splits, we present the first assessment under cell-blind and drug-blind cross-validation.

# 3. Supplementary References

[1] E. Wang, S. Schmidgall, P.F. Jaeger, F. Zhang, R. Pilgrim, Y. Matias, J. Barral, D. Fleet, S. Azizi, TxGemma: Efficient and Agentic LLMs for Therapeutics, (2025). https://arxiv.org/pdf/2504.06196 (accessed May 27, 2025).

[2] C. Wang, G.A. Kumar, J.C. Rajapakse, Drug discovery and mechanism prediction with explainable graph neural networks, Scientific Reports 2024 15:1 15 (2025) 1–14. https://doi.org/10.1038/s41598-024-83090-3.

[3] F. Taj, L.D. Stein, MMDRP: drug response prediction and biomarker discovery using multi-modal deep learning, Bioinformatics Advances 4 (2024). https://doi.org/10.1093/BIOADV/VBAE010.

[4] S. Park, E. Silva, A. Singhal, M.R. Kelly, K. Licon, I. Panagiotou, C. Fogg, S. Fong, J.J.Y. Lee, X. Zhao, R. Bachelder, B.A. Parker, K.T. Yeung, T. Ideker, A deep learning model of tumor cell architecture elucidates response and resistance to CDK4/6 inhibitors, Nature Cancer 2024 5:7 5 (2024) 996–1009. https://doi.org/10.1038/s43018-024-00740-1.

[5] C. Lao, P. Zheng, H. Chen, Q. Liu, F. An, Z. Li, DeepAEG: a model for predicting cancer drug response based on data enhancement and edge-collaborative update strategies, BMC Bioinformatics 25 (2024) 1–16. https://doi.org/10.1186/S12859-024-05723-8/TABLES/3.

[6] C. Belli, A. Sagingalieva, M. Kordzanganeh, N. Kenbayev, D. Kosichkina, T. Tomashuk, A. Melnikov, Hybrid Quantum Neural Network For Drug Response Prediction, (2023). https://doi.org/10.3390/cancers15102705.

[7] D.E. Hostallero, L. Wei, L. Wang, J. Cairns, A. Emad, Preclinical-to-clinical Anti-cancer Drug Response Prediction and Biomarker Identification Using TINDL, Genomics Proteomics Bioinformatics 21 (2023) 535–550. https://doi.org/10.1016/J.GPB.2023.01.006.

[8] K. Sada Del Real, A. Rubio, Discovering the mechanism of action of drugs with a sparse explainable network, EBioMedicine 95 (2023) 104767. https://doi.org/10.1016/j.ebiom.2023.104767.

[9] L. Jiang, C. Jiang, X. Yu, R. Fu, S. Jin, X. Liu, DeepTTA: a transformer-based model for predicting cancer drug response, Brief Bioinform 23 (2022). https://doi.org/10.1093/BIB/BBAC100.

[10] Z. Zuo, P. Wang, X. Chen, L. Tian, H. Ge, D. Qian, SWnet: a deep learning model for drug response prediction from cancer genomic signatures and compound chemical structures, BMC Bioinformatics 22 (2021) 1–16. https://doi.org/10.1186/S12859-021-04352-9/TABLES/4.

[11] I. Jin, H. Nam, HiDRA: Hierarchical Network for Drug Response Prediction with Attention, J Chem Inf Model 61 (2021) 3858–3867. https://doi.org/10.1021/ACS.JCIM.1C00706/SUPPL_FILE/CI1C00706_SI_003.XLSX.

[12] X. Huang, K. Huang, T. Johnson, M. Radovich, J. Zhang, J. Ma, Y. Wang, ParsVNN: parsimony visible neural networks for uncovering cancer-specific and drug-sensitive genes and pathways, NAR Genom Bioinform 3 (2021). https://doi.org/10.1093/NARGAB/LQAB097.

[13] Q. Liu, Z. Hu, R. Jiang, M. Zhou, DeepCDR: a hybrid graph convolutional network for predicting cancer drug response, Bioinformatics 36 (2020) i911–i918. https://doi.org/10.1093/BIOINFORMATICS/BTAA822.

[14] B.M. Kuenzi, J. Park, S.H. Fong, J.F. Kreisberg, J. Ma, Predicting Drug Response and Synergy Using a Deep Learning Model of Human Cancer Cells, (2020). https://doi.org/10.1016/j.ccell.2020.09.014.

[15] L. Deng, Y. Cai, W. Zhang, W. Yang, B. Gao, H. Liu, Pathway-guided deep neural network toward interpretable and predictive modeling of drug sensitivity, J Chem Inf Model 60 (2020) 4497–4505. https://doi.org/10.1021/ACS.JCIM.0C00331/SUPPL_FILE/CI0C00331_SI_001.PDF.

[16] M. Manica, A. Oskooei, J. Born, V. Subramanian, J. Saéz-Rodríguez, M. Rodríguez Martínez, Toward Explainable Anticancer Compound Sensitivity Prediction via Multimodal Attention-Based Convolutional Encoders, Mol Pharm (2019) 4797–4806. https://doi.org/10.1021/ACS.MOLPHARMACEUT.9B00520/SUPPL_FILE/MP9B00520_SI_001.PDF.

[17] H. Sharifi-Noghabi, O. Zolotareva, C.C. Collins, M. Ester, MOLI: multi-omics late integration with deep neural networks for drug response prediction, Bioinformatics 35 (2019) i501–i509. https://doi.org/10.1093/BIOINFORMATICS/BTZ318.

[18] U.T. Shankavaram, W.C. Reinhold, S. Nishizuka, S. Major, D. Morita, K.K. Chary, M.A. Reimers, U. Scherf, A. Kahn, D. Dolginow, J. Cossman, E.P. Kaldjian, D.A. Scudiero, E. Petricoin, L. Liotta, J.K. Lee, J.N. Weinstein, Transcript and protein expression profiles of the NCI-60 cancer cell panel: An integromic microarray study, Mol Cancer Ther 6 (2007) 820–832. https://doi.org/10.1158/1535-7163.MCT-06-0650/357219/P/TRANSCRIPT-AND-PROTEIN-EXPRESSION-PROFILES-OF-THE.

[19] J. Barretina, G. Caponigro, N. Stransky, K. Venkatesan, A.A. Margolin, S. Kim, C.J. Wilson, J. Lehár, G. V. Kryukov, D. Sonkin, A. Reddy, M. Liu, L. Murray, M.F. Berger, J.E. Monahan, P. Morais, J. Meltzer, A. Korejwa, J. Jané-Valbuena, F.A. Mapa, J. Thibault, E. Bric-Furlong, P. Raman, A. Shipway, I.H. Engels, J. Cheng, G.K. Yu, J. Yu, P. Aspesi, M. De Silva, K. Jagtap, M.D. Jones, L. Wang, C. Hatton, E. Palescandolo, S. Gupta, S. Mahan, C. Sougnez, R.C. Onofrio, T. Liefeld, L. MacConaill, W. Winckler, M. Reich, N. Li, J.P. Mesirov, S.B. Gabriel, G. Getz, K. Ardlie, V. Chan, V.E. Myer, B.L. Weber, J. Porter, M. Warmuth, P. Finan, J.L. Harris, M. Meyerson, T.R. Golub, M.P. Morrissey, W.R. Sellers, R. Schlegel, L.A. Garraway, The Cancer Cell Line Encyclopedia enables predictive modelling of anticancer drug sensitivity, Nature 483 (2012) 603–607. https://doi.org/10.1038/NATURE11003.

[20] F. Iorio, T.A. Knijnenburg, D.J. Vis, G.R. Bignell, M.P. Menden, M. Schubert, N. Aben, E. Gonçalves, S. Barthorpe, H. Lightfoot, T. Cokelaer, P. Greninger, E. van Dyk, H. Chang, H. de Silva, H. Heyn, X. Deng, R.K. Egan, Q. Liu, T. Mironenko, X. Mitropoulos, L. Richardson, J. Wang, T. Zhang, S. Moran, S. Sayols, M. Soleimani, D. Tamborero, N. Lopez-Bigas, P. Ross-Macdonald, M. Esteller, N.S. Gray, D.A. Haber, M.R. Stratton, C.H. Benes, L.F.A. Wessels, J. Saez-Rodriguez, U. McDermott, M.J. Garnett, A Landscape of Pharmacogenomic Interactions in Cancer, Cell 166 (2016) 740–754. https://doi.org/10.1016/J.CELL.2016.06.017/ATTACHMENT/2690C3CA-4270-4D2A-8984-FDDCBFA738C7/MMC9.PDF.

[21] R. Shi, J. Sun, Z. Zhou, M. Shi, X. Wang, Z. Gao, T. Zhao, M. Li, Y. Shu, Integration of multiple machine learning approaches develops a gene mutation-based classifier for accurate immunotherapy outcomes, Npj Precision Oncology 2025 9:1 9 (2025) 1–16. https://doi.org/10.1038/s41698-025-00842-8.

[22] Y. Li, D.E. Hostallero, A. Emad, Interpretable deep learning architectures for improving drug response prediction performance: myth or reality?, Bioinformatics 39 (2023). https://doi.org/10.1093/BIOINFORMATICS/BTAD390.

[23] M. Mamunur Rashid, K. Selvarajoo, Advancing drug-response prediction using multi-modal and -omics machine learning integration (MOMLIN): a case study on breast cancer clinical data, Brief Bioinform 25 (2024) bbae300. https://doi.org/10.1093/BIB/BBAE300.

[24] F. Firoozbakht, B. Yousefi, O. Tsoy, J. Baumbach, B. Schwikowski, Comparative evaluation of feature reduction methods for drug response prediction, Scientific Reports 2024 14:1 14 (2024) 1–11. https://doi.org/10.1038/s41598-024-81866-1.

[25] S. Munquad, A.B. Das, Uncovering the subtype-specific disease module and the development of drug response prediction models for glioma, Heliyon 10 (2024). https://doi.org/10.1016/J.HELIYON.2024.E27190.

[26] R.J.A. Fijneman, E. van den Broek, S. van Lieshout, C. Rausch, B. Ylstra, M.A. van de Wiel, G.A. Meijer, S. Abeln, GeneBreak: detection of recurrent DNA copy number aberration-associated chromosomal breakpoints within genes, F1000Res 5 (2017) 2340. https://doi.org/10.12688/F1000RESEARCH.9259.2.

[27] X. An, X. Chen, D. Yi, H. Li, Y. Guan, Representation of molecules for drug response prediction, Brief Bioinform 23 (2022) 1–11. https://doi.org/10.1093/BIB/BBAB393.

[28] M. Krenn, Q. Ai, S. Barthel, N. Carson, A. Frei, N.C. Frey, P. Friederich, T. Gaudin, A.A. Gayle, K.M. Jablonka, R.F. Lameiro, D. Lemm, A. Lo, S.M. Moosavi, J.M. Nápoles-Duarte, A.K. Nigam, R. Pollice, K. Rajan, U. Schatzschneider, P. Schwaller, M. Skreta, B. Smit, F. Strieth-Kalthoff, C. Sun, G. Tom, G. Falk von Rudorff, A. Wang, A.D. White, A. Young, R. Yu, A. Aspuru-Guzik, SELFIES and the future of molecular string representations, Patterns 3 (2022). https://doi.org/10.1016/J.PATTER.2022.100588.

[29] K. Huang, C. Xiao, L. Glass, J. Sun, Explainable Substructure Partition Fingerprint for Protein, Drug, and More, (n.d.).

[30] B. Zagidullin, Z. Wang, Y. Guan, E. Pitkänen, J. Tang, Comparative analysis of molecular fingerprints in prediction of drug combination effects, Brief Bioinform 22 (2021) 1–15. https://doi.org/10.1093/BIB/BBAB291.

[31] H. Liu, W. Peng, W. Dai, J. Lin, X. Fu, L. Liu, L. Liu, N. Yu, Improving anti-cancer drug response prediction using multi-task learning on graph convolutional networks, Methods 222 (2024) 41–50. https://doi.org/10.1016/J.YMETH.2023.11.018.
